# Systemic Interplay of BDNF and Serotonin Pathways Defines Behavioral and Molecular Responses to Midbrain 5-HT7 Overexpression and Chronic Ethanol Consumption

**DOI:** 10.3390/biom16010106

**Published:** 2026-01-08

**Authors:** Alexander Rodnyy, Alina Oreshko, Dmitry Eremin, Vladimir Naumenko, Darya Bazovkina

**Affiliations:** Federal Research Center Institute of Cytology and Genetics, Siberian Division of the Russian Academy of Science, Novosibirsk 630090, Russia; shaburovaas@bionet.nsc.ru (A.O.); eremin@bionet.nsc.ru (D.E.); naumenko2002@bionet.nsc.ru (V.N.); drinterf@bionet.nsc.ru (D.B.)

**Keywords:** 5-HT_7_ receptor, chronic alcoholization, AAV-mediated overexpression, male mice, brain 5-HT system, Brain-derived neurotrophic factor (BDNF)

## Abstract

Chronic ethanol exposure and genetic factors interact to drive neuroadaptations in alcohol use disorders (AUD). However, the system-level coordination of molecular responses across brain regions remains unclear. The 5-HT system and BDNF are key regulators of neuroplasticity in alcoholism. The 5-HT_7_ receptor modulates both behavior and serotonin signaling. We investigated midbrain 5-HT_7_ overexpression in C57BL/6 mice given 5-week ethanol access. Our results showed complex, region-specific changes in 5-HT and BDNF signaling, as well as selective behavioral alterations. Ethanol abolished the antidepressant-like effect of 5-HT_7_ overexpression and increased anxiety-like behavior, without affecting baseline locomotion or novel object recognition. At the molecular level, ethanol suppressed 5-HT_7_-mediated CREB/BDNF signaling and differentially regulated 5-HT_1A_ and 5-HT_2A_ expression across regions. To extract general principles, we used integrative systems analysis based on population-averaged generalized estimating equations (GEE), and mapped effects in the (t_1_, t_2_) plane. We identified two regularities: first, regional specificity of responses, and second, divergence across regulatory levels, with opposing effects more frequent at the mRNA level and concordant effects more common at the protein level. These findings suggest that neuroadaptation to combined 5-HT_7_ and ethanol factors follows region- and level-specific rules, rather than a single global program, underscoring the value of integrative analysis.

## 1. Introduction

*The epigraph*

*“A not well-discussed property of data: it is toxic in large quantities”*

*Nassim Nicholas Taleb, Antifragile: Things That Gain from Disorder*

Chronic alcohol consumption exerts a destructive impact on the central nervous system, disrupting neuroplasticity, cognitive functions, and behavioral regulation. According to the World Health Organization (2022), alcohol abuse is responsible for 3 million deaths annually, accounting for 5.3% of all global mortality. Central mechanisms underlying alcohol-induced pathogenesis include dysfunction of the neurotransmitter systems and imbalance of neurotrophic factors. These disturbances impair synaptic transmission and form the basis for cognitive deficits, affective disorders, and loss of the brain’s adaptive capacity under conditions of chronic alcohol exposure [1].

Among the numerous molecular cascades involved in the pathogenesis of alcohol dependence, Brain-derived neurotrophic factor (BDNF) attracts particular attention as key regulator of neuroplasticity and neuronal survival. Under physiological conditions, BDNF supports neurogenesis, synaptic plasticity, and neuronal viability via activation of the TrkB receptor. In contrast, its precursor, proBDNF, can exert opposite effects by activating the p75 receptor and inducing apoptosis and long-term synaptic depression [2,3]. Chronic alcohol exposure disrupts the balance between BDNF and proBDNF: mature BDNF levels are reduced in the raphe nuclei and hippocampus, correlating with memory impairment and cognitive dysfunction [4,5,6]. Concurrently, increased levels of proBDNF and p75 receptor expression are observed, particularly in brain regions responsible for emotional regulation and addiction-related behavior, such as the prefrontal cortex, amygdala, and raphe nuclei. This imbalance in the BDNF/proBDNF system promotes neurodegenerative processes and impairs adaptive behavior [6].

The serotonergic system also plays a critical role in regulating behavior and neuroplasticity in the context of alcoholism. Serotonin (5-HT) is involved in the control of impulsivity, emotional processing, and the development of addictive behaviors. While short-term alcohol intake may transiently elevate serotonin levels, chronic alcohol exposure leads to serotonergic system depletion, which is associated with cognitive and emotional impairments [7,8].

Despite the substantial body of evidence on the role of the serotonergic system in the pathogenesis of alcohol dependence, the specific functions of individual serotonin receptors, including the 5-HT_7_ receptor, remain insufficiently understood. In recent years, the 5-HT_7_ receptor has emerged as a promising therapeutic target for the correction of neuropsychiatric disorders due to its unique ability to modulate neuroplasticity, circadian rhythms, emotional behavior, and cognitive processes. These effects are mediated by mechanisms involving increased intracellular cAMP levels and activation of adenylate cyclase [9,10,11,12].

Studies have demonstrated that 5-HT_7_ receptors play a significant role in the development of alcohol and drug addiction [10]. These receptors are expressed in key brain structures involved in addictive behavior, including the ventral tegmental area, nucleus accumbens, amygdala, hippocampus, and prefrontal cortex. Pharmacological evidence suggests that selective 5-HT_7_ receptor antagonists can reduce alcohol and drug intake, as well as attenuate compulsive behaviors associated with addiction. However, such systemic manipulations do not resolve where within the distributed serotonergic circuitry 5-HT_7_ signaling is functionally decisive or which cell types mediate these effects. Moreover, chronic alcohol consumption leads to increased expression of 5-HT_7_ receptors in certain brain regions, such as the hippocampus and striatum, potentially contributing to the development and reinforcement of addictive behaviors [10], whereas the causal role of 5-HT_7_ signaling in the serotonergic source nuclei of the midbrain raphe remains less directly tested.

In this context, the 5-HT_7_ receptor becomes an attractive target for modulating alcohol-induced states, offering novel avenues for therapeutic intervention. Collectively, these observations highlight the multifactorial nature of alcohol’s neurobiological effects and reinforce the need for integrative approaches in both research and treatment strategies for alcohol use disorders.

Particular interest lies in the cross-talk between the serotonergic system and BDNF. It has been shown that serotonin and BDNF can mutually regulate each other’s expression and function. For instance, BDNF enhances the activity of serotonergic neurons and increases the expression of 5-HT receptors, while serotonin modulates the synthesis and release of BDNF [13]. Under chronic alcohol exposure, alterations in the BDNF system may further exacerbate serotonergic dysfunction, thereby amplifying cognitive and behavioral impairments—and vice versa. Investigation of the interplay between the brain serotonergic system and BDNF under conditions of chronic alcohol exposure offers new insights into the neurobiological mechanisms underlying alcohol dependence and associated disorders, and suggests these systems as a promising target for therapeutic development. However, most modern neurobiological research focuses on individual markers, which, to some extent, limits the ability to understand the systemic picture of neuroadaptations and the overall picture. The complex interactions between factors and brain regions remain poorly understood. In recent years, systematic approaches have been increasingly used to solve this problem. For example, gene co-expression analysis (WGCNA) revealed gene modules associated with anxiety during alcoholism in mice [14]. Other scientists successfully integrate multiomic data to search for new molecular targets related to predisposition to alcohol abuse [15]. These approaches demonstrate the great value of multidimensional analysis for understanding pathogenesis of alcohol use disorders.

In this study, we focus on the midbrain as a key structure for investigating serotonergic modulation of alcohol-associated disturbances for several reasons. First, the midbrain contains the dorsal and median raphe nuclei—the principal sources of serotonergic projections to multiple brain regions, including the limbic system, prefrontal cortex, and hippocampus [16]. These projections are critically involved in the regulation of emotional states, cognitive functions, and the development of addiction. Second, the midbrain houses the ventral tegmental area (VTA), a central component of the brain reward system directly implicated in the mechanisms of alcohol dependence [17]. Third, previous research has shown that midbrain serotonergic neurons are particularly sensitive to chronic alcohol exposure, exhibiting pronounced changes in functional activity and receptor expression, including 5-HT_7_ receptor [6].

Localized overexpression of 5-HT_7_ receptors in this region provides a unique opportunity to assess their modulatory influence on ascending serotonergic pathways projecting to limbic and cortical areas, and to examine the associated behavioral and molecular effects under chronic alcohol exposure. To this extent, we employed AAV-mediated overexpression of 5-HT_7_ receptors in midbrain neurons of mice followed by chronic ethanol consumption. This approach enables a more precise evaluation of the contribution of these receptors to the pathogenesis of alcohol-induced cognitive impairments.

Thus, the aim of this study is to investigate the behavioral and molecular effects of 5-HT_7_ receptor overexpression in midbrain neurons under conditions of chronic alcohol consumption in mice. We applied an integrated approach combining visualization of effects at the system level (based on Ordinary Least Squares (OLS) projections) and robust statistical modeling (using GEE) in order to simultaneously assess the influence of the type of molecule, brain region, functional system and effect magnitude on the nature of the response.

## 2. Materials and Methods

### 2.1. Animals

Male C57BL/6 mice (8–10 weeks; 25 ± 1 g, n = 65) were obtained from the SPF (Specific Pathogen-Free) vivarium of the Federal Research Center, Institute of Cytology and Genetics SB RAS (Novosibirsk, Russia) (supported by the basic research projects No. FWNR-2026-0028 and RFMEFI62117X0015). After weaning, mice were housed in groups of five per cage under standard conditions (14:10 h light/dark, 20–22 °C, humidity 45–50%, food/water ad libitum). All surgeries were performed under appropriate anesthesia, and every effort was made to minimize animal suffering.

### 2.2. Production of rAAV Vectors

The cDNA encoding mouse *Htr7* was subcloned into the pAAV-Syn-EGFP vector. Recombinant adeno-associated viral (rAAV) particles were generated by co-transfection of HEK293 cells with either pAAV-Syn-Htr7-EGFP or control pAAV-Syn-EGFP plasmids, together with AAV-DJ and pHelper plasmids (Cell Biolabs, Inc., San Diego, CA, USA). Viral particles were collected 48 h post-transfection according to the protocol described by Grimm et al. (2003) [18]. The viral genome copy number was determined by real-time quantitative PCR using specific primers (Forward: 5′-cctggttgctgtctctttatgagg; Reverse: 5′-tgacaggtggtggcaatgc), with a standard curve generated from serial dilutions of a plasmid of known concentration. All viral preparations had comparable genomic titers, averaging 1 × 10^9^ viral genomes per microliter.

### 2.3. Stereotaxic Microinjections

Mice were anesthetized by intraperitoneally (i.p.) administered a solution (1 mL/kg) of 2,2,2-Tribromoethanol (T48402-25G, Sigma-Aldrich, Darmstadt, Germany) in 2-Methyl-2-butanol (240486, Sigma-Aldrich, Darmstadt, Germany). The AAV vectors (carrying pAAV-Syn-HTR7-EGFP or pAAV-Syn-EGFP plasmids) were unilaterally injected into the raphe nuclei area using following coordinates: AP-3; L-2; DV-4; Angle = 38°; Rotation = 40°, according to the mouse brain atlas [19] and our previous study [20]. 1 uL of the viral vector (1 × 10^9^ viral particles/μL) were microinjected into site at the rate of 0.2 μL/min using a Hamilton Syringe. The syringe was left in place for 5 min and then raised slowly. Histological validation of injection targeting was performed in a separate validation animal as described in our previous article [20]; representative EGFP expression/spread and the targeting schematic are provided in Supplementary Figure S2. Brains from the main experimental cohort were used for protein/RNA analyses, precluding histological verification in all animals.

### 2.4. Experimental Design

The study used a 2 × 2 factorial design: AAV vector (EGFP control vs. 5-HT7-EGFP) × drinking regimen (water vs. 10% ethanol). On day 0, mice received stereotaxic injections of the genetic construct; from day 1 they had single-bottle access to 10% (*v*/*v*) ethanol or water for 5 weeks. C57BL/6 mice are known to exhibit a strong preference for ethanol over water when given a choice between two bottles across a broad range of ethanol concentrations [21,22]. Chronic alcohol exposure continued until the animals were decapitated, followed by dissection of brain regions on ice, including the prefrontal cortex, hippocampus, hypothalamus, and the midbrain raphe nuclei area.

**Figure 1:**
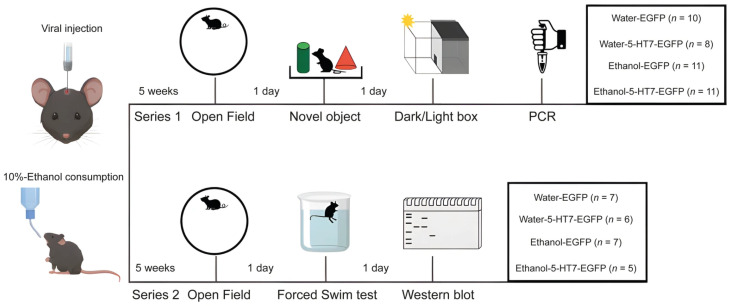
Experimental design and timeline. The validation (atlas-based targeting schematic with stereotaxic coordinates and approach parameters and representative EGFP expression/spread in the midbrain raphe region) is shown in Figure S2.

Two independent experimental series were conducted. In each experiment, mice were divided into four groups: Water-EGFP, Water-5-HT7-EGFP, Ethanol-EGFP, and Ethanol-5-HT7-EGFP. Mice of the first series (n = 40) were tested in the open field, novel object and dark-light box tests before sacrifice. Mice of the second series (n = 25) were tested in the open field and forced swim tests before sacrifice. Two days before testing, animals were weighed and single-housed. The experimental design and the exact number of mice in each group are presented in Figure 1.

### 2.5. Behavioral Tests

#### 2.5.1. Open Field Test (OF)

The open field test was conducted using a circular arena (40 cm in diameter) surrounded by a white plastic wall (25 cm in height) and illuminated from below through a matt semi-transparent floor by two 12 W halogen lamps positioned 40 cm beneath the arena floor. Each mouse was placed near the wall, and its behavior was recorded for 5 min using a digital video camera (Sony, Japan) located 80 cm from the arena. The arena was cleaned after each test session. Video footage was analyzed frame by frame using custom EthoStudio software 2.0. Horizontal locomotor activity (path length) was automatically measured.

#### 2.5.2. Novel Object Recognition Test (NOR)

This test relies on differential exploration time of novel versus familiar objects. It was conducted over three days, following previously described procedures [23,24]. The same circular arena as in the open field test was used. On the first day, each mouse was habituated to the empty arena for 10 min. On the second day, two identical, previously unfamiliar objects were placed symmetrically relative to the center of the arena, and the mouse was allowed to explore them for 10 min. On the third day, one of the familiar objects was replaced with a novel object differing in shape, material, or color, and the exploration time of both objects was recorded for 10 min. Object exploration was defined as the mouse approaching, sniffing, or making physical contact with the object using its snout or forepaws. Recognition memory was assessed by calculating the ratio of time spent exploring the novel object to the total time spent exploring both objects.

#### 2.5.3. Dark-Light Box Test (DLB)

This test was conducted using a plastic chamber (20 × 40 × 27 cm), painted white and divided into two compartments—light and dark—by an opaque partition with a 7 × 7 cm opening. The light compartment had a translucent plastic floor illuminated from below by two 12 W halogen lamps. Mice were placed in the light compartment, facing the opening. Over a 5 min session, the following parameters were recorded: the duration of time spent in the light compartment, the proportion of the light area explored, and the number of entries into the light compartment from the dark.

#### 2.5.4. Forced Swim Test (FST)

Each mouse was placed in a transparent glass cylinder (30 cm in diameter and height) filled with water at 25 °C. After a 2 min habituation period, the animal’s mobility was recorded automatically for 4 min using custom EthoStudio software. The program measured the rate of change in the animal’s silhouette, defined as the number of pixels associated with the animal that changed between consecutive frames [25].

### 2.6. RT-PCR in Real Time

Total RNA was isolated with Trizol reagent (ThermoScientific, Waltham, MA, USA) and 1 µg of the mRNA was used for cDNA synthesis with a random hexanucleotide primer. PCR was conducted as in our previous works [20,26,27]. Real-time quantitative PCR was performed for following genes: *Polr2a*, *Htr1a*, *Htr7*, *Htr2a*, *Tph2*, *Slc6a4*, *Bdnf*, *Ntrk2*, *Ngfr*, *Creb1*. Primers’ parameters are presented in Table 1.

Gene transcription was presented as the relative number of cDNA copies with respect to 100 copies of *Polr2a* cDNA [28,29,30].

### 2.7. Western Blot

The Western blotting analysis was conducted as in our previous works [6,20]. In brief, to determine the protein levels of 5-HT_1A_, 5-HT_2A_, 5-HT_7_, TrkB, p75 receptors and 5-HTT in brain regions, membrane protein fraction was prepared from brain tissue. For assessment of TPH2, BDNF, proBDNF, CREB, pCREB cytosol protein fraction was used. Then samples were separated by 10% or 15% SDS-PAGE and transferred to nitrocellulose membranes. The membranes were probed with primary antibodies (Table 2).

For protein detection, membranes were incubated with secondary antibody conjugated with HRP (anti-rabbit IgG for the primary antibodies, 1:10,000, Thermo Fisher Scientific Cat# G-21234 or anti-mouse 1:30,000, Waltham, MA, USA). The bands were visualized using Clarity Western ECL Substrate (#1705061, Bio-Rad Laboratories, Hercules, CA, USA) and the C-DiGit Blot Scanner (LI-COR, Lincoln, NE, USA). Quantification of protein bands was performed by ImageStudio 5.2 (LI-COR Image Studio Software, RRID:SCR_015795, USA). Target protein levels were normalized to GAPDH levels and represented as the percentage of control animals.

5-HT_7_–EGFP fusion protein (~79 kDa) was visualized in the midbrain with anti-EGFP antibodies, because ten commercial anti-5-HT_7_ antibodies failed specificity validation on 5-HT_7_ knockout tissue (for validation [31]), and were non-informative in our previous work [20]. Accordingly, anti-5-HT_7_ immunoblotting in the present material was not used to draw conclusions about receptor abundance. Viral overexpression was robust at the transcript level (≈100-fold by qPCR), and injection targeting/EGFP fluorescence in the raphe region is documented in Supplementary Figure S2. This part is presented in more detail in the discussion of the article.

### 2.8. Statistics and Data Representation

Statistical analyses and data visualisation were performed in GraphPad Prism 9.0 and Python 3.9 (statsmodels 0.14.4, scipy 1.12.0, matplotlib 3.9.4). Unless noted otherwise, results are reported as mean ± 95% CI. The statistical significance was set at α = 0.05.

Per-target analysis (ANOVA). For individual target measurements, group differences were assessed with a two-way ANOVA (factors: Overexpression, Alcohol, and their interaction). Significant main effects or interactions were followed by Fisher’s least-significant-difference (LSD) test. Residual normality was confirmed with the Kolmogorov–Smirnov and Shapiro–Wilk tests; outliers were removed using the ROUT test.

System-level exploratory analysis (OLS visualization). To visualize system-wide regulatory patterns, we fitted an ordinary least squares (OLS) model for each target–region pair:

Y_i_ = β_0_ + β_1_·Alcohol + β_2_·Overexpression + ε_i_,

where Y_i_ denotes either transcript abundance or normalized protein level.

The resulting coefficient t-statistics (t_1_ = β_1_/SE(β_1_) and t_2_ = β_2_/SE(β_2_)) were plotted in a two-dimensional plane. This visualization served an exploratory purpose, illustrating the direction and relative statistical confidence of each factor’s effect across the entire dataset [32,33].

System-level hypothesis testing (GEE). To formally test the patterns observed in the exploratory analysis, we employed Generalized Estimating Equations (GEE), with a binomial logit link to obtain population-averaged effects and correctly account for within-gene clustering across regions (i.e., repeated measurements for the same gene) [34]. A similar approach for clustering neurobiological data has been used by Bender et al., 2013, Neurobiology of Disease [35].

The binary outcome variable was EffectDirection, defined as 1 for same-direction (synergistic) effects (Quadrants I and III) and 0 for opposite-direction (antagonistic) effects (Quadrants II and IV). Predictors included: molecular DataType, brain Region, functional GeneSystem, and the effect Magnitude (Euclidean norm of t-statistics). Reference levels for categorical variables were set to Protein (for DataType), Frontal Cortex (for Region), and BDNF pathway (for GeneSystem).

The primary model specification for inference used Gene (N = 22 unique marker names) as the clustering unit with an Exchangeable working correlation structure. This conservative approach was chosen to ensure robust standard error estimation given the data structure. To confirm the stability of our findings, we pre-planned and conducted two sensitivity analyses: (i) using an Independence working correlation, and (ii) using a Generalized Linear Model (GLM) with cluster-robust standard errors grouped by biologically unified GeneEntity (N = 10 clusters, for example *Htr1a* + 5-HT_1A_, or *Bdnf* + mBDNF + proBDNF + proBDNF/mBDNF).

Two-stage analysis of quadrant patterns. To further explore the nature of antagonistic interactions, a secondary GEE was fitted exclusively on observations within Quadrants II and IV to identify which factors predict the predominance of Overexpression antagonism (Q-II) versus Alcohol antagonism (Q-IV). In this model, the outcome was a binary variable distinguishing Quadrant II (coded as 1) from Quadrant IV (coded as 0).

All models used robust (sandwich) standard errors. Effects are reported as Odds Ratios (OR) with 95% Confidence Intervals (CI).

## 3. Results

### 3.1. Behavior

**Figure 2:**
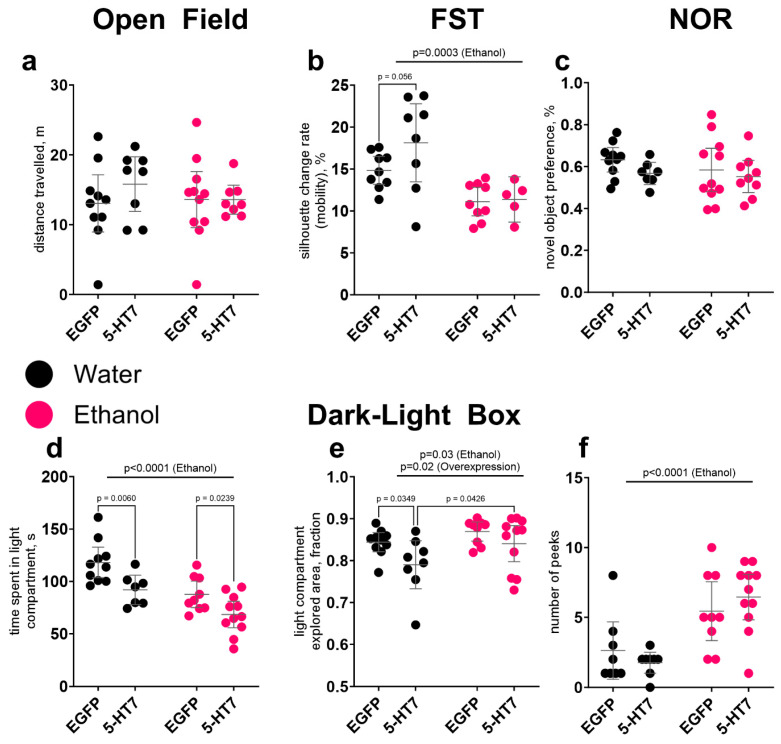
The effect of chronic ethanol consumption and midbrain overexpression of 5-HT_7_ receptor on behavior. The following were evaluated: (**a**) general locomotor activity in the Open Field test; (**b**) mobility in the forced swimming test (FST) as an indicator of depressive-like behavior; (**c**) novel object preference in the Novel Object Recognition (NOR) test; (**d**–**f**) anxiety level in the Dark-Light box test, measured as (**d**) time spent in the light compartment, (**e**) the explored area of the light compartment, and (**f**) the number of peeks. The data is presented as individual values for each animal, displaying the mean ± 95% CI. Statistical analysis was performed using two-factor ANOVA (factors: chronic Ethanol, 5-HT_7_ receptor overexpression in midbrain). The *p*-values for significant main effects are shown at the top of the panels. The *p*-values for the posteriori Fisher test (LSD), showing significant differences between individual groups, are indicated separately. N = 5–11 animals in the group. Detailed statistical output of the per-target two-way ANOVAs is available in Table S2(c). Lines without brackets indicate main effects (two-way model); bracketed lines indicate pairwise post hoc comparisons.

No significant effects of 5-HT_7_ overexpression, chronic alcohol exposure, or their interaction on total traveled distance were observed in the OF and NOR tests (Figure 2a,c). However, in the FST, ethanol exposure significantly reduced mobility (F_1,27_ = 17.37, *p* = 0.0003), while 5-HT_7_ overexpression led to a trend-level increase in this parameter in water-drinking mice (*p* = 0.056) (Figure 2b).

Notable changes were observed in the DLB test (Figure 2d–f). Both ethanol exposure (F_1,33_ = 20.11, *p* < 0.0001) and 5-HT_7_ overexpression (F_1,33_ = 14.16, *p* = 0.0007) significantly reduced the time spent in the light compartment. Additionally, these factors also influenced the explored area of the light compartment, but in different ways (F_1,34_ = 5.09, *p* = 0.03 and F_1,34_ = 6.02, *p* = 0.02, respectively). Specifically, the explored area in the 5-HT_7_-water group was significantly lower than in the EGFP-water (*p* = 0.03) and 5-HT_7_-ethanol (*p* = 0.04) groups, suggesting that chronic alcohol exposure restored the reduced level of this parameter under conditions of 5-HT_7_ overexpression. Another measured parameter, the number of head peeks, was significantly increased by chronic ethanol consumption (F_1,33_ = 24.10, *p* < 0.0001), while 5-HT_7_ overexpression failed to affect it.

### 3.2. Expression of 5-HT Brain System Key Elements

**Figure 3:**
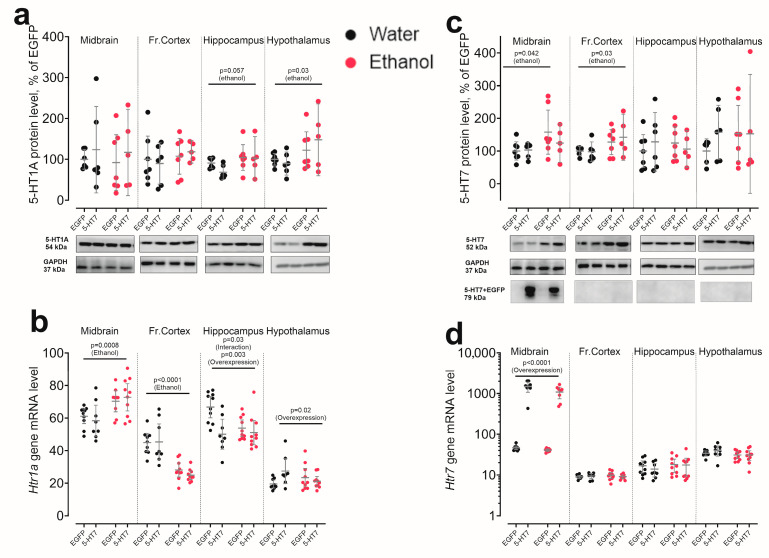
The effect of chronic ethanol consumption and midbrain overexpression of 5-HT_7_ receptor on the mRNA and protein levels of key components of the brain 5-HT system. The following were evaluated for 5-HT_1A_ receptor (**a**,**b**), the 5-HT_7_ receptor (**c**,**d**). Protein data is normalized to GAPDH and presented as a percentage of the control group (EGFP, water). Gene transcription is presented as the number of the gene cDNA copies with respect to 100 cDNA copies of *Polr2a*. The data is presented as individual values for each animal, displaying the mean ± 95% CI. Statistical analysis was performed using two-factor ANOVA (factors: chronic Ethanol, 5-HT_7_ receptor overexpression in midbrain). The *p*-values for significant main effects are shown at the top of the panels. The *p*-values for the posteriori Fisher test (LSD), showing significant differences between individual groups, are indicated separately. N = 5–11 animals in the group. Detailed statistical output of the per-target two-way ANOVAs is available in Table S1(a,b). Lines without brackets indicate main effects (two-way model); bracketed lines indicate pairwise post hoc comparisons. Original Western Blots images for Figure 3 are found in Supplementary Figures.

**Figure 4:**
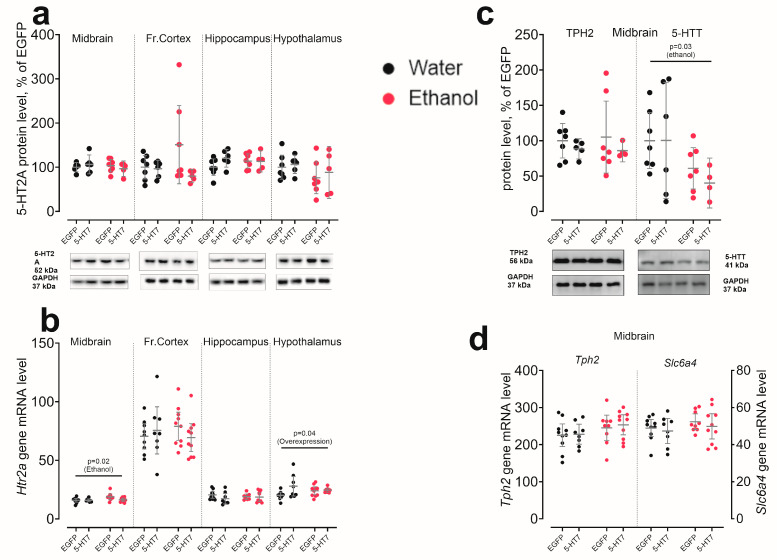
The effect of chronic ethanol consumption and midbrain overexpression of 5-HT_7_ receptor on the mRNA and protein levels of key components of the brain 5-HT system. The following were evaluated for the 5-HT_2A_ receptor (**a**,**b**), as well as tryptophan hydroxylase 2 (TPH2/*Tph2*) and the serotonin transporter (5-HTT/*Slc6a4*) (**c**,**d**). Protein data is normalized to GAPDH and presented as a percentage of the control group (EGFP, water). Gene transcription is presented as the number of the gene cDNA copies with respect to 100 cDNA copies of *Polr2a*. The data is presented as individual values for each animal, displaying the mean ± 95% CI. Statistical analysis was performed using two-factor ANOVA (factors: chronic Ethanol, 5-HT_7_ receptor overexpression in midbrain). The *p*-values for significant main effects are shown at the top of the panels. The *p*-values for the posteriori Fisher test (LSD), showing significant differences between individual groups, are indicated separately. N = 5–11 animals in the group. Detailed statistical output of the per-target two-way ANOVAs is available in Table S1(a,b). Lines without brackets indicate main effects (two-way model); bracketed lines indicate pairwise post hoc comparisons. Original Western Blots images for Figure 4 are found in Supplementary Figures.

Analysis of brain serotonin system components at the mRNA and protein levels revealed gene-specific and region-specific effects of ethanol and 5-HT_7_ receptor gene overexpression (Figure 3 and Figure 4).

As expected, AAV injection produced an approximately 100-fold increase in *Htr7* mRNA in the midbrain compared with AAV-Syn-EGFP controls (F_1,29_ = 124.50, *p* < 0.0001), but did not affect *Htr1a*, *Htr2a*, *Tph2*, or *Slc6a4* mRNA levels in this region (all *p* > 0.05). No detectable changes in *Htr7* mRNA were observed in the frontal cortex, hippocampus, or hypothalamus (all *p* > 0.05). At the protein level, Western blotting with commercial anti-5-HT_7_ antibodies yielded a 5-HT_7_–immunoreactive signal that showed an ethanol-related increase in the midbrain (F_1,20_ = 4.66, *p* = 0.043) and frontal cortex (F_1,20_ = 5.17, *p* = 0.034); however, given the lack of validated 5-HT_7_ antibody specificity (Ref. [31]) this signal cannot be interpreted as a reliable measure of 5-HT_7_ receptor abundance and may reflect cross-reactivity. Consistently, viral 5-HT_7_ overexpression did not produce a detectable change in this anti-5-HT_7_ immunoreactive signal in any analyzed region. No significant ethanol × overexpression interactions were detected for the anti-5-HT_7_ signal or other serotonin-related proteins in the midbrain (all *p* > 0.05) (Figure 3c,d).

Ethanol produced opposite changes in *Htr1a* mRNA across regions. In the midbrain, ethanol increased *Htr1a* gene transcription (F_1,33_ = 13.49, *p* = 0.001), whereas in the frontal cortex it consistently decreased *Htr1a* mRNA (F_1,33_ = 49.65, *p* < 0.0001). In the hippocampus, ethanol did not alter *Htr1a* mRNA, but 5-HT_7_ receptor gene overexpression reduced *Htr1a* mRNA (F_1,34_ = 9.18, *p* = 0.005) and produced a significant ethanol × overexpression interaction (F_1,34_ = 5.13, *p* = 0.030). In the hypothalamus, 5-HT_7_ receptor gene overexpression increased *Htr1a* mRNA, specifically in the water-drinking group (F_1,20_= 5.61, *p* = 0.023), while ethanol had no effect. Alcohol exposure significantly increased 5-HT_1A_ protein level in the hypothalamus (F_1,34_ = 4.80, *p* = 0.040), whereas no significant changes were detected in the midbrain, frontal cortex, or hippocampus (Figure 3a,b).

For *Htr2a* mRNA, ethanol selectively increased transcription in the midbrain (F_1,33_ = 5.48, *p* = 0.02), with no significant ethanol effect in other regions. In contrast, 5-HT_7_ receptor gene overexpression modestly increased *Htr2a* mRNA only in the hypothalamus (F_1,31_ = 4.31, *p* = 0.046) while leaving other regions unaffected. Across all regions, 5-HT_2A_ protein levels did not show significant changes in response to either ethanol or 5-HT_7_ receptor gene overexpression (Figure 4a,b).

In the midbrain, neither ethanol nor 5-HT_7_ receptor gene overexpression altered *Tph2* or *Slc6a4* mRNA levels. Consistently, TPH2 protein was unaffected by either factor (Figure 4c). In contrast, 5-HTT protein showed a selective ethanol effect: chronic ethanol reduced 5-HTT levels in the midbrain (F_1,20_ = 5.46, *p* = 0.030), with no significant ethanol × overexpression interaction (Figure 4d).

Overall, ethanol exposure produced detectable gene- and region-specific modulation of the serotonin system, with primary effects on *Htr1a, Htr2a*, 5-HT_7_, and 5-HTT. Notably, these effects were generally more consistent at the transcriptional level than at the protein level, and several endpoints showed transcription–protein discordance. 5-HT_7_ receptor gene overexpression exerted a strong, targeted effect on *Htr7* transcription in the midbrain and selectively modulated *Htr1a* and *Htr2a* mRNAs in hippocampal and hypothalamic circuits, lowering *Htr1a* in the hippocampus, while showing an opposite-direction effect in the hypothalamus, and increasing *Htr2a* in the hypothalamus while having limited consequences at the protein level. Notably, 5-HT_7_ receptor gene overexpression did not produce measurable effects on gene transcription or protein expression in the frontal cortex.

Overall, the results suggest selective and region-specific effects of ethanol exposure and 5-HT_7_ overexpression, which are mostly modest in magnitude and do not uniformly propagate from transcription to protein expression on serotonin-related gene and protein expression, particularly in the midbrain and frontal cortex, while 5-HT_7_ overexpression shows a limited but significant impact on *Htr7* mRNA transcription. These findings highlight the complexity of ethanol’s modulation of the serotonin system and the potential contributions of 5-HT_7_ receptor activity in this process.

### 3.3. Expression of BDNF Related Elements

Analysis of BDNF-related components at the mRNA and protein levels revealed gene- and region-specific effects of ethanol and midbrain 5-HT_7_ receptor gene overexpression across the frontal cortex, hippocampus, hypothalamus, and midbrain. Key targets included *Bdnf* (proBDNF and mBDNF), *Ngfr* (p75 receptor), *Ntrk2* (TrkB receptor), and *Creb1* (CREB transcription factor).

**Figure 5:**
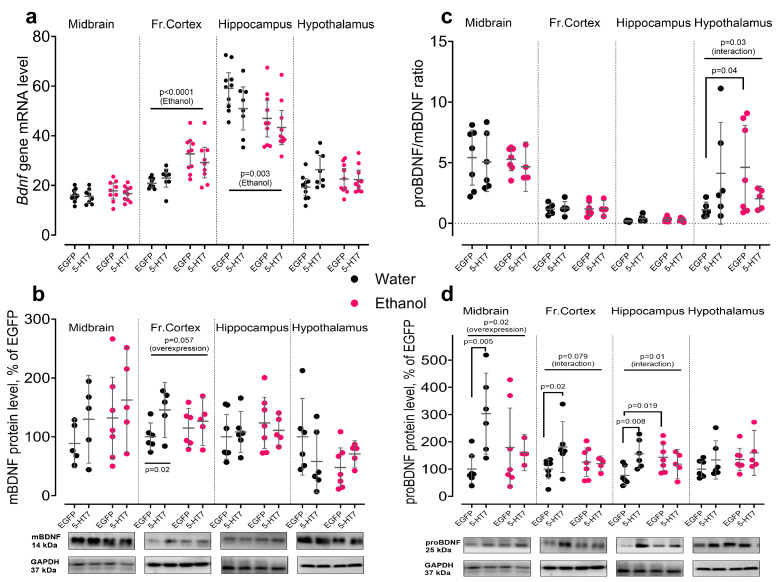
Changes in the BDNF expression under the influence of chronic ethanol exposure and midbrain overexpression of 5-HT_7_ receptor. The panels show changes in the levels of *Bdnf* gene transcription and mature BDNF protein and (**a**,**b**), proBDNF (**c**) and proBDNF/mBDNF ratio (**d**). For each target, data on the level of protein (Western blotting) and/or the corresponding transcript are presented (quantitative PCR) in four studied brain regions. Protein levels are normalized to GAPDH and are presented as a percentage of the control group (EGFP, water). Each gene contains data on the level of protein (Western blotting) and the corresponding transcript (qPCR) in the midbrain, frontal cortex, hippocampus, and hypothalamus. Protein data is normalized to GAPDH and presented as a percentage of the control group (EGFP, water). Gene transcription is presented as the number of the gene cDNA copies with respect to 100 cDNA copies of *Polr2a*. The data is presented as individual values for each animal, displaying the mean ± 95% CI. Statistical analysis was performed using two-factor ANOVA (factors: chronic Ethanol, 5-HT_7_ receptor overexpression in midbrain). The *p*-values for significant main effects are shown at the top of the panels. The *p*-values for the posteriori Fisher test (LSD), showing significant differences between individual groups, are indicated separately. N = 5–11 animals in the group. Detailed statistical output of the per-target two-way ANOVAs is available in Table S1(a,b). Lines without brackets indicate main effects (two-way model); bracketed lines indicate pairwise post hoc comparisons. Original Western Blots images for Figure 5 are found in Supplementary Figures.

Ethanol bidirectionally regulated *Bdnf* mRNA along the rostro–caudal axis. In the frontal cortex, ethanol increased *Bdnf* gene transcription (F_1,32_ = 21.25, *p* < 0.0001), whereas in the hippocampus it decreased *Bdnf* mRNA (F_1,34_ = 9.87, *p* = 0.003). In the midbrain, *Bdnf* mRNA was not significantly affected (*p* > 0.05), and no ethanol or overexpression effects on *Bdnf* mRNA were detected in the hypothalamus (Figure 5a). At the protein level, 5-HT_7_ receptor gene overexpression increased proBDNF in the midbrain (F_1,32_ = 5.96, *p* = 0.024), while leaving mature BDNF unchanged (*p* > 0.05). In the frontal cortex, overexpression increased BDNF protein (F_1,18_ = 4.54, *p* = 0.047) and showed a trend toward higher proBDNF (F_1,18_ = 3.39, *p* = 0.079). In the hippocampus, an ethanol × overexpression interaction was detected for proBDNF levels (F_1,20_ = 7.32, *p* = 0.014), whereas BDNF itself was unchanged. In the hypothalamus, BDNF and proBDNF protein levels were not significantly altered (Figure 5b,d), but a significant ethanol × overexpression interaction was observed for the proBDNF/BDNF ratio (F_1,20_ = 5.47, *p* = 0.030), indicating region-specific modulation of BDNF processing (Figure 5c).

**Figure 6:**
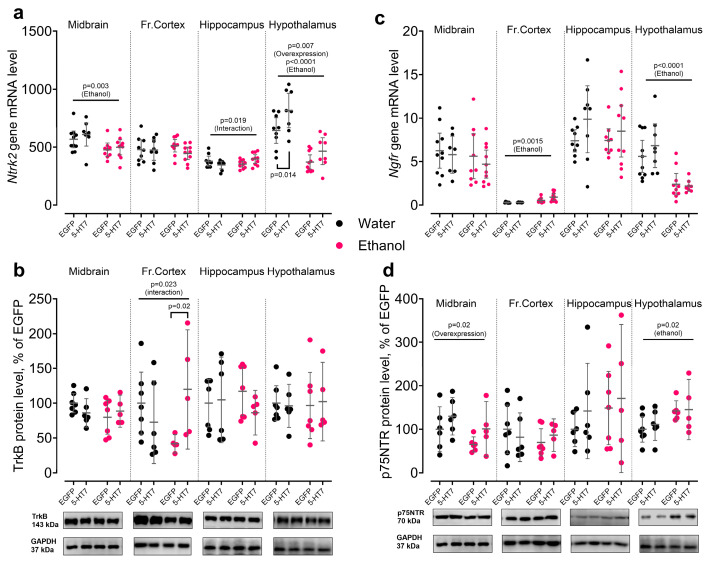
Changes in the components of the BDNF signaling pathway under the influence of chronic ethanol exposure and midbrain overexpression of 5-HT_7_ receptor. The panels show changes in the levels of *Ntrk* gene transcription and TrkB receptor protein (**a**,**b**), *Ngfr* gene expression and p75 receptor protein (**c**,**d**). For each target, data on the level of protein (Western blotting) and/or the corresponding transcript are presented (quantitative PCR) in four studied brain regions. Protein levels are normalized to GAPDH and are presented as a percentage of the control group (EGFP, water). Each gene contains data on the level of protein (Western blotting) and the corresponding transcript (qPCR) in the midbrain, frontal cortex, hippocampus, and hypothalamus. Protein data is normalized to GAPDH and presented as a percentage of the control group (EGFP, water). Gene transcription is presented as the number of the gene cDNA copies with respect to 100 cDNA copies of *Polr2a*. The data is presented as individual values for each animal, displaying the mean ± 95% CI. Statistical analysis was performed using two-factor ANOVA (factors: chronic Ethanol, 5-HT_7_ receptor overexpression in midbrain). The *p*-values for significant main effects are shown at the top of the panels. The *p*-values for the posteriori Fisher test (LSD), showing significant differences between individual groups, are indicated separately. N = 5–11 animals in the group. Detailed statistical output of the per-target two-way ANOVAs is available in Table S1(a,b). Lines without brackets indicate main effects (two-way model); bracketed lines indicate pairwise post hoc comparisons. Original Western Blots images for Figure 6 are found in Supplementary Figures.

*Ntrk2* mRNA showed notable region-specific sensitivity to ethanol. In the midbrain, ethanol decreased *Ntrk2* gene transcription (F_1,33_ = 9.73, *p* = 0.004). In the hypothalamus, ethanol also reduced *Ntrk2* mRNA (F_1,33_ = 43.71, *p* < 0.0001), and 5-HT_7_ receptor gene overexpression additionally modulated *Ntrk2* gene transcription in this region (F_1,33_ = 7.98, *p* = 0.007). In the frontal cortex, *Ntrk2* mRNA was not significantly affected (*p* > 0.05), whereas in the hippocampus, *Ntrk2* mRNA level was regulated by an ethanol × overexpression interaction (F_1,34_ = 6.05, *p* = 0.019), without clear main effects of either factor (Figure 6a). At the protein level, TrkB was largely stable across regions: no significant main effects were detected in the midbrain, hippocampus, or hypothalamus (all *p* > 0.05). In the frontal cortex, however, an ethanol × overexpression interaction was observed for TrkB protein (F_1,19_ = 6.03, *p* = 0.024), indicating that combined exposure modulates TrkB signaling specifically in this region (Figure 6b).

*Ngfr* mRNA responded consistently to ethanol in forebrain regions. In the frontal cortex, ethanol increased *Ngfr* gene transcription (F_1,28_ = 12.10, *p* = 0.002), while in the hypothalamus it produced the opposite effect (F_1,32_= 27.81, *p* < 0.0001). In contrast, *Ngfr* mRNA remained unchanged in the midbrain and hippocampus (both *p* > 0.05) (Figure 6c). At the protein level, 5-HT_7_ receptor gene overexpression increased p75 in the midbrain (F_1,18_ = 6.55, *p* = 0.020), whereas ethanol increased p75 in the hypothalamus (F_1,20_ = 6.52, *p* = 0.019). No significant effects on p75 were detected in the frontal cortex or hippocampus, and no ethanol × overexpression interactions were observed for p75 (Figure 6d).

**Figure 7:**
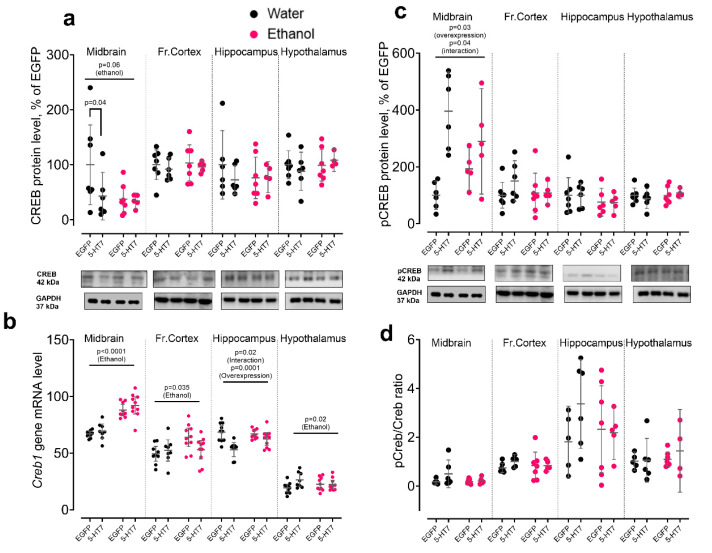
The effect of chronic ethanol exposure and midbrain 5-HT_7_ overexpression on the expression and phosphorylation of the transcription factor CREB. The panels show changes in the levels of total CREB protein (**a**), *Creb1* gene transcription (**b**), pCREB phosphorylated form (**c**), and pCREB/CREB ratio reflecting the degree of activation (**d**). For each target, data on the level of protein (Western blotting) and/or the corresponding transcript are presented (quantitative PCR) in four studied brain regions. Protein levels are normalized to GAPDH and are presented as a percentage of the control group (EGFP, water). Each gene contains data on the level of protein (Western blotting) and the corresponding transcript (qPCR) in the midbrain, frontal cortex, hippocampus, and hypothalamus. Protein data is normalized to GAPDH and presented as a percentage of the control group (EGFP, water). Gene transcription is presented as the number of the gene cDNA copies with respect to 100 cDNA copies of *Polr2a*. The data is presented as individual values for each animal, displaying the mean ± 95% CI. Statistical analysis was performed using two-factor ANOVA (factors: chronic Ethanol, 5-HT_7_ receptor overexpression in midbrain). The *p*-values for significant main effects are shown at the top of the panels. The *p*-values for the posteriori Fisher test (LSD), showing significant differences between individual groups, are indicated separately. N = 5–11 animals in the group. Detailed statistical output of the per-target two-way ANOVAs is available in Table S1(a,b). Lines without brackets indicate main effects (two-way model); bracketed lines indicate pairwise post hoc comparisons. Original Western Blots images for Figure 7 are found in Supplementary Figures.

Ethanol modestly but consistently upregulated *Creb1* mRNA in several regions. In the midbrain, *Creb1* gene transcription was strongly increased (F_1,32_ = 75.06, *p* < 0.0001), and in the frontal cortex ethanol also elevated *Creb1* mRNA (F_1,33_ = 4.83, *p* = 0.035). In the hypothalamus, ethanol increased *Creb1* mRNA (F_1,33_ = 6.06, *p* = 0.02). In the hippocampus, *Creb1* mRNA was primarily affected by 5-HT_7_ receptor gene overexpression: it reduced *Creb1* mRNA in water-drinking animals (F_1,34_ = 17.33, *p* = 0.0002), with a significant ethanol × overexpression interaction (F_1,34_ = 5.53, *p* = 0.02) (Figure 7b). Notably, this transcriptional increase was not accompanied by detectable changes in total CREB protein, indicating decoupling between transcription and protein abundance under the present conditions. At the protein level, midbrain pCREB was increased by 5-HT_7_ overexpression (F_1,19_ = 4.98, *p* = 0.03), whereas ethanol showed only a trend toward reduced total CREB (F_1,21_ = 3.94, *p* = 0.06). No significant CREB or pCREB changes were detected in the other regions (Figure 7a,c,d).

Overall, ethanol exposure selectively modulated *Bdnf, Ngfr, Ntrk2*, and *Creb1* transcription in a gene- and region-specific manner, with particularly prominent effects in the frontal cortex and hypothalamus. Protein-level regulation involved proBDNF, BDNF, p75, TrkB, and pCREB, including several ethanol × overexpression interactions that point to nonlinear integration of neurotrophic and serotonergic signaling. In contrast, the effects of 5-HT_7_ overexpression were more selective, targeting midbrain proBDNF, p75, and pCREB and modifying *Ntrk2* and *Creb1* transcription in specific forebrain regions.

### 3.4. Integrative Analysis of Treatment Effects Across Brain Regions

After conducting and analyzing the results of a two-factor ANOVA for each mRNA/protein expression value in all the studied regions, we set a goal to summarize the directions and strength of effects across the entire data set, including 34 “gene-region” pairs for mRNA and 42 “protein-region pairs”. To visualize global regulatory patterns, we applied an approach that is ideologically similar to projection methods on latent structures such as PLS and O-PLS, which have proven themselves well for analyzing complex and noisy data [32]. In particular, we used a projection in the plane of t-statistics obtained from OLS models for each endpoint [33]. This integrative visualization is intended as an exploratory, descriptive summary of directionality across endpoints rather than a standalone inference of biological mechanism. Therefore, quadrant assignments are interpreted together with effect sizes and the transcription–protein correspondence for each target.

**Figure 8:**
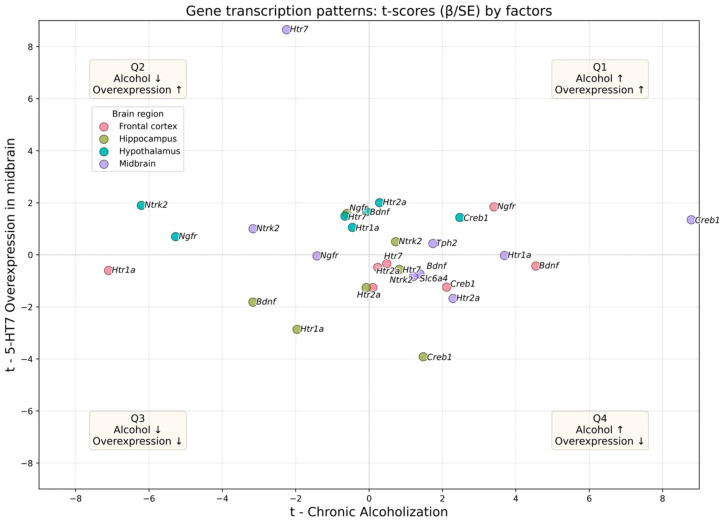
Transcript-level effects visualized in the (t_1_, t_2_) plane. The t_1_—t axis is the coefficient statistic for chronic Alcoholization; the t_2_—t axis is the coefficient statistic for 5-HT_7_ receptor overexpression. Each point is a unique target × region pair. The color encodes a region of the brain; the dotted lines t = 0 define the boundaries of the quadrants. Interpretation of the quadrants: I (t_1_ > 0, t_2_ > 0) is collinear enhancement; II (t_1_ < 0, t_2_ > 0)—alcohol ↓, 5-HT_7_ ↑; III (t_1_ < 0, t_2_ < 0) is collinear reduction; IV (t_1_ > 0, t_2_ < 0)—alcohol ↑, 5-HT_7_ ↓. The captions next to the dots are the names of the analyzed genes. Note: the further away the point is from the origin, the higher the modulus of the effect (|t|). Per-target OLS model coefficients used for system-level visualization are available in Table S3.

**Figure 9:**
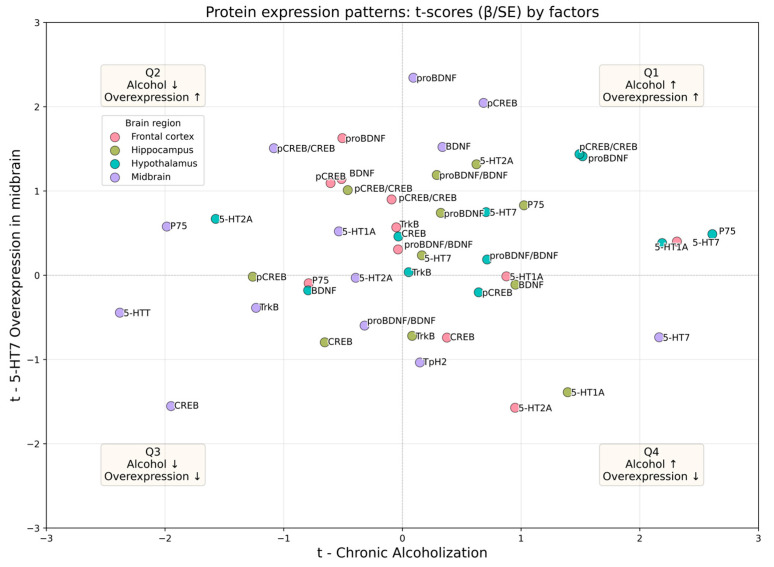
Protein-level effects visualized in the (t_1_, t_2_) plane. The t_1_—t axis is the coefficient statistic for chronic Alcoholization; the t_2_—t axis is the coefficient statistic for 5-HT_7_ receptor overexpression. Each point is a unique target × region pair. The color encodes a region of the brain; the dotted lines t = 0 define the boundaries of the quadrants. Interpretation of the quadrants: I (t_1_ > 0, t_2_ > 0) is collinear enhancement; II (t_1_ < 0, t_2_ > 0)—alcohol ↓, 5-HT_7_ ↑; III (t_1_ < 0, t_2_ < 0) is collinear reduction; IV (t_1_ > 0, t_2_ < 0)—alcohol ↑, 5-HT_7_ ↓. The captions next to the dots are the names of the analyzed genes. Note: the further away the point is from the origin, the higher the modulus of the effect (|t|). Per-target OLS model coefficients used for system-level visualization are available in Table S4.

In the scatter plots shown (Figure 8 and Figure 9), the x-axis (t_1_) represents the effect magnitude of chronic ethanol exposure, while the y-axis (t_2_) reflects the effect of midbrain 5-HT_7_ overexpression. This approach allows distinguishing robust, significant effects (points distant from the origin) from variability unrelated to the experimental factors (points near the center) and classifying the interaction type by quadrant. Owing to the balanced 2 × 2 factorial design, the t-statistics derived from the OLS models offer inferential power equivalent to that of ANOVA, while providing an intuitive system-level visualization of regulatory patterns. Quadrant interpretation: I (t_1_ > 0, t_2_ > 0)—concordant increase; II (t_1_ < 0, t_2_ > 0)—opposing pattern (ethanol ↓, 5-HT_7_ ↑); III (t_1_ < 0, t_2_ < 0)—concordant decrease; IV (t_1_ > 0, t_2_ < 0)—opposing pattern (ethanol ↑, 5-HT_7_ ↓).

The distribution across quadrants differed between mRNA and protein levels: at the mRNA level, opposing effects predominated (QII + QIV = 64.7%; QIV 38.2%, QII 26.5%, QI 17.6%, QIII 17.6%), whereas at the protein level, concordant regulation was more frequent (QI + QIII = 54.7%; QI 35.7%, QII 26.2%, QIII 19.0%, QIV 19.0%).

Regional patterns also diverged: for mRNA, the frontal cortex was dominated by QIV-type responses (~75%), the hypothalamus by QII (~75%), and the hippocampus by QIII (~50%); for proteins, the hypothalamus showed enrichment in QI (~70%). A formal comparison of distributions across quadrants was non-significant (χ^2^(3) = 4.74, *p* = 0.19), although this result should be interpreted with caution since the χ^2^ test ignores data clustering and effect magnitude.

To quantitatively assess the observed patterns and identify factors determining the tendency toward synergistic or antagonistic regulation, we applied a population-averaged logistic GEE model [34], which accounts for clustered, non-independent observations and allows simultaneous evaluation of molecule type, brain region, and functional system affiliation.

The model clustered by molecular marker (GENE = 22) was selected as the primary and most conservative specification, providing the most reliable assessment of fixed effects. The main model identified several robust predictors and confirmed two principal regulatory patterns (Table 3).

**Figure 10:**
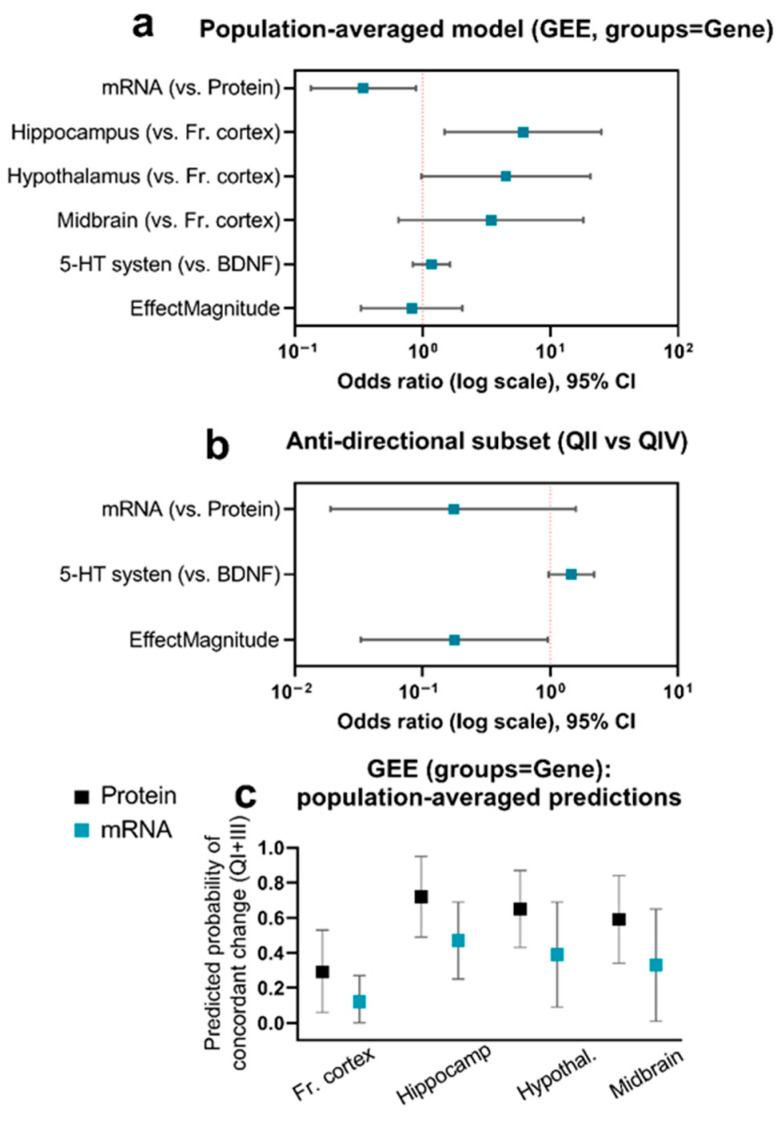
System-level analysis of regulatory patterns using population-averaged models. (**a**) Main GEE model results (OR and 95% CI) for the probability of concordant regulation. (**b**) Two-stage model results identifying predictors for the QII vs. QIV pattern within antagonistic responses; a system-level predictor shifts the balance toward QIV. (**c**) Model-predicted probabilities (from panel (**a**)) of concordant regulation for proteins and mRNAs across the examined brain regions. Vertical dashed line at OR = 1 in panels (**a**,**b**). Key predictors effects across different model specifications are available in Figure S1.

First, a significant discrepancy emerged between transcriptional (mRNA) and post-translational (protein) levels (Figure 10a). The odds of concordant (synergistic) regulation for mRNA were only 34% of those for protein (OR = 0.34, 95% CI [0.13, 0.89]; *p* = 0.027), indicating a predominance of opposing, potentially compensatory mechanisms at the transcriptional level.

Second, the analysis revealed marked regional specificity. Relative to the frontal cortex, the hippocampus exhibited a stronger tendency toward synergistic regulation (OR = 6.12; 95% CI [1.49, 25.12]; *p* = 0.012), with a similar but non-significant trend for the hypothalamus (OR = 4.49; 95% CI [0.98, 20.53]; *p* = 0.053). No significant effects were detected for the midbrain (*p* > 0.05). In contrast, neither functional system affiliation (5-HT vs. BDNF-related components) nor effect magnitude significantly influenced the probability of synergistic regulation in this specification.

To further characterize antagonistic interactions, we conducted a two-stage analysis restricted to opposing effects (Quadrants II and IV). This model assessed factors determining the predominance of one antagonistic pattern over the other: “alcohol ↓, 5-HT_7_ ↑” (QII) versus “alcohol ↑, 5-HT_7_ ↓” (QIV).

The analysis revealed a significant effect of the functional system. For components of the 5-HT system, the odds of exhibiting the QII pattern were markedly lower than those for the QIV pattern (OR ≈ 0.18; 95% CI [0.03, 0.96]; *p* = 0.044), indicating that antagonistic regulation within the serotonergic network predominantly follows the “alcohol activation/overexpression suppression” mode (QIV) (Figure 10b).

Predicted probabilities from the main GEE model (Figure 10c) further visualize these relationships: proteins consistently show higher probabilities of concordant regulation than mRNAs across all regions, and the hippocampus (and, to a lesser extent, the hypothalamus) displays higher predicted probabilities than the cortex. Sensitivity and robustness analyses confirmed the stability of these findings. Equivalent results were obtained when using an alternative correlation structure (independence) and a GLM with cluster-robust standard errors by GeneEntity; the direction and magnitude of key effects remained unchanged. Full coefficient tables are provided in the Supplementary file (Table S5).

## 4. Discussion

The 5-HT_7_ receptor is a promising target for the treatment of alcohol use disorders (AUD). Human genetic studies link polymorphisms in *HTR7* gene to increased risk of alcohol dependence. In preclinical models, blocking 5-HT_7_ receptors modulates alcohol intake: high-affinity 5-HT_7_ antagonists reduce ethanol consumption in alcohol-preferring mice and rats and attenuate alcohol-seeking and craving [10]. In this study, we investigate the role of 5-HT_7_ receptors by producing of AAV-mediated overexpression of 5-HT_7_ receptors in the midbrain in the condition of chronic ethanol consumption.

Beyond AUD, 5-HT_7_ receptor has become a focus in psychiatry. Several modern antidepressants and antipsychotics, including vortioxetine and lurasidone, bind 5-HT_7_ with high affinity; part of their efficacy, particularly rapid antidepressant effects and cognitive enhancement, may arise from 5-HT_7_ inhibition [36]. Within AUD, modulation of 5-HT_7_ receptor activity can affect multiple pathogenic links: restoring serotonergic balance, reducing anxiety and depressive symptoms, and potentially weakening alcohol’s reinforcing properties. Targeting 5-HT_7_ receptor signaling is therefore a plausible avenue for new therapeutics. Here, we used AAV-mediated overexpression of 5-HT_7_ receptors in the midbrain, a hub of reward circuitry critical for alcohol intake and dependence, where the ventral tegmental area and allied midbrain structures undergo adaptations that assign emotional salience to alcohol-related cues, which promotes dependence [37]. Despite an ~100-fold increase in *Htr7* transcription in the target tissue, downstream behavioral and molecular effects are modest. This pattern may reflect tissue and cell-type heterogeneity, post-transcriptional regulation, and circuit-level homeostatic compensation. While *Htr7* transcriptional overexpression was robust at the tissue level, variability in local spread/cell-type targeting cannot be fully excluded and may contribute to modest downstream effects.

Chronic ethanol exposure in mice induces pronounced behavioral alterations: heightened anxiety, depression-like states, and cognitive deficits [38,39]. These effects arise in part from serotonergic dysregulation [40,41]. Both inhibition and localized enhancement of 5-HT_7_ receptor function can yield antidepressant- and anxiolytic-like effects. The selective 5-HT_7_ receptor antagonist SB-269970 reduces immobility in the forced swim test (FST) and increases exploration in the open arms of the elevated plus maze (EPM). Likewise, *Htr7* knockout mice show reduced immobility, which is consistent with an antidepressant-like phenotype [36]. In our previous work, AAV-mediated 5-HT_7_ receptor overexpression in the midbrain of genetically predisposed ASC mice increased locomotor activity in the open field (OF) and reduced immobility in the FST; in C57BL/6 mice it improved FST performance without altering baseline locomotion [20].

In the present study, this antidepressant-like effect was replicated in water-drinking C57BL/6 controls, but chronic ethanol exposure abolished it and markedly reduced FST mobility. Ethanol did not change OF locomotion or novel object recognition (NOR) performance. In the light–dark box (LDB), a paradigm that pits exploratory drive against avoidance of brightly lit, potentially threatening spaces, both 5-HT_7_ receptor overexpression and ethanol consumption increased anxiety-like behavior. Notably, ethanol significantly elevated the frequency of risk-assessment peeks into the light compartment, whereas 5-HT_7_ receptor overexpression did not alter this measure. More peeks together with less time in the illuminated compartment may indicate heightened vigilance and cautious exploration, that is, an anxious yet goal-directed strategy [42,43]. As emphasized by La-Vu et al. (2020), rodent anxiety tests capture the balance between curiosity-driven exploration and threat avoidance [44]. The observed profile fits a cautious exploratory state, characterized by information gathering while minimizing exposure to risk.

Chronic ethanol exposure alters not only behavioral patterns but also reshapes gene expression networks in various brain regions. These changes at the level of genetic expression correlate with increased ethanol consumption and are enriched for genes involved in synaptic transmission and plasticity [45,46]. Gene networks involving genes encoding the neurotrophin BDNF and elements of the brain 5-HT system play a central role in these adaptations [47,48].

Many of the statistically significant transcriptional effects observed here are modest in magnitude. We interpret these small shifts not as large-scale remodeling, but as subtle biasing or priming of regulatory programs within heterogeneous tissue, where cell type composition and baseline expression levels constrain the dynamic range. Such effects are most informative when they are gene- and region-specific, directionally aligned with the primary perturbation, and accompanied by convergent evidence at another level (protein expression or behaviour). When transcriptional shifts are not mirrored at the protein level, we treat them as suggestive and discuss plausible decoupling mechanisms, including post-transcriptional regulation, temporal lags, and differences in measurement sensitivity.

It has been previously shown that in the dorsal raphe nuclei, where the cell bodies of serotonin neurons are located, prolonged ethanol increases *Htr7* gene mRNA while reducing protein levels of tryptophan hydroxylase-2 (TPH2), the rate-limiting enzyme for serotonin synthesis. Paradoxically, despite the reduction in protein abundance, TPH2 enzymatic activity rises [6]. In our experiment, *Tph2* gene transcription did not change significantly. Ethanol, however, altered serotonin transporter (5-HTT) protein without affecting the *Slc6a4* mRNA. 5-HT_7_ receptor gene overexpression also did not influence these measures. Results from previous studies reflect these region-specific trends. Acute ethanol exposure resulted in a transient increase in 5-HT levels in the nucleus accumbens, while there was a progressive loss of serotonergic axons and a global decrease in 5-HTT binding; this effect was most pronounced in early-onset alcoholism and resulted in a hyposerotonergic state [41].

According to most of the literature, the effects of chronic ethanol consumption on 5-HT receptors are region-specific and sometimes opposite. Thus, it was shown that chronic ethanol exposure was associated with upregulation of the 5-HT_1A_ receptor in the cerebral cortex, hippocampus, and amygdala, although the direction and speed of the effect varied depending on the region [41]. Previously, we did not find an effect of ethanol on the 5-HT_1A_ receptor, but we did find a significant decrease in 5-HT_2A_ receptor protein in the frontal cortex [6]. In the present experiment, 5-HT_1A_ receptor protein increased in the hippocampus and hypothalamus, and *Htr1a* gene mRNA increased in the midbrain and decreased in the frontal cortex. Meanwhile, *Htr1a* mRNA levels increased in the hypothalamus and decreased in the hippocampus in mice drinking water while overexpressing the 5-HT_7_ receptor gene. Notably, ethanol suppressed these shifts. A similar interaction was observed for *Htr2a* mRNA levels in the hypothalamus, while *Htr2a* mRNA levels in the midbrain increased only in mice consuming ethanol. Finally, ethanol increased 5-HT_7_-immunoreactive signal (anti-5-HT_7_) in the midbrain and frontal cortex, while changes in *Htr7* mRNA levels were limited to the midbrain (the site of AAV overexpression action). We failed to detect the elevation of 5-HT_7_ receptor protein levels in the midbrain of mice receiving the target gene construct due to the low specificity of available antibodies. We have discussed this situation in detail previously, but in short, there are currently no successful commercially available antibodies to the 5-HT_7_ receptor [20].

It is well established that BDNF and the 5-HT system form a tightly coupled regulatory network. There is evidence that neurotrophins (BDNF and GDNF) stimulate growth of serotonergic neurons and upregulate *Tph2*, *Htr1a*, and *Htr2a*, whereas serotonin, acting through its receptors and CREB, induces BDNF expression across multiple brain regions [13]. It is also known that prolonged ethanol intake produces marked alterations in the BDNF system: *BDNF* is reduced (mRNA level in the midbrain and protein level in the hippocampus), while proBDNF is increased in several regions, including midbrain, cortex, and amygdala [6]. These shifts point to a diversion of neurotrophic signaling toward a maladaptive regime, with proBDNF/p75 pathways that dampen neuronal survival and synaptic plasticity prevailing over canonical mature BDNF/TrkB signaling.

In our experiment we showed that elements of this network can nonetheless respond in a compensatory manner, with *Bdnf* mRNA levels rising in the frontal cortex and, conversely, declining in the hippocampus. At the protein level, the pattern diverges, consistent with post-translational control. Mature BDNF level increased in the frontal cortex in water-drinking mice under 5-HT_7_ receptor overexpression condition; this increase was absent with ethanol-consuming animals, indicating suppression of 5-HT_7_ receptor-mediated effects by ethanol. The same directionality, more pronounced and spanning midbrain, frontal cortex, and hippocampus, was observed for proBDNF level. Interpreting TrkB and p75 receptor expression is complicated by regional mismatches with BDNF or proBDNF level changes. Since BDNF exerts its positive influence through TrkB receptors and its negative influence through p75 receptors, the following changes mediated by the action of ethanol can be considered negative: a decrease in the mRNA of TrkB receptor gene in the midbrain, as well as an increase in the level of mRNA of the p75 receptor gene in the frontal cortex and the protein of this receptor in the hypothalamus. Moreover, the now familiar ethanol-dependent suppression of AAV effects was evident for *Ntrk2* mRNA level in the hypothalamus.

CREB provides a molecular intersection between serotonergic and BDNF-related signaling. It is activated via the cAMP/PKA cascade in response to 5-HT_7_ receptor stimulation and, in parallel, via MAPK following TrkB receptor activation by BDNF. Since 5-HT_7_ receptors are Gs-coupled, their activation elevates cAMP and can enhance CREB activity and BDNF expression [49,50]. In our data, CREB phosphorylation was significantly increased in the midbrain in 5-HT_7_ receptor-overexpression groups, consistent with previous reports. Chronic ethanol also increased *Creb1* mRNA level in midbrain, frontal cortex, and hypothalamus. This is consistent with data showing that acute ethanol activates cAMP/PKA cascade and upregulates some CREB-dependent genes, particularly in hippocampus and thalamus [51]. Notably, in our experiment, increased expression of the 5-HT_7_ receptor gene reduced total CREB protein levels in the midbrain of water-drinking mice, whereas no reduction was observed following ethanol exposure. This result further emphasizes that ethanol consumption suppressed the effects mediated by AAV.

Taken together, the 5-HT_7_ receptor → cAMP/PKA → CREB → BDNF axis functions as a finely balanced module; ethanol perturbs this balance, plausibly driving maladaptive circuit remodeling that underlies altered neuroplasticity and behavior.

In this study, we performed a comprehensive, system-level analysis of molecular changes induced by chronic ethanol exposure and by 5-HT_7_ receptor overexpression. We showed that the joint effect of these factors, whether synergistic or antagonistic, depends on two determinants: the neuroanatomical context and the molecular level of regulation. The most consistent pattern in our analysis is pronounced regional specificity. The hippocampus exhibits a higher probability of concordant changes, and the hypothalamus shows a similar tendency, albeit weaker. This pattern aligns with the high plasticity of these structures and their sensitivity to ethanol [52,53] and to modulation of 5-HT_7_ receptor-dependent pathways [54].

A second key observation is a divergence between transcriptional and post-translational levels. Statistically, opposing effects are more frequent at the mRNA level, whereas concordant effects prevail at the protein level. This is consistent with strong compensatory mechanisms, in which transcriptional shifts are partially buffered by post-translational regulation to maintain homeostasis [55,56].

A more detailed analysis uncovered a specific mechanism of antagonistic regulation within the serotonergic system. Although membership in the 5-HT system was not a significant predictor in the primary conservative model, the two-stage analysis revealed an informative pattern: when antagonistic effects arise in this system, they occur disproportionately in Quadrant IV, that is, as “alcohol activation with overexpression suppression.” This subtle but statistically significant result is consistent with the possibility of complex cross-regulation. Chronic alcohol stimulation likely triggers compensatory processes that reduce the functional impact of 5-HT_7_ receptor-dependent pathways, particularly when these pathways are in excess.

Methodological constraints should be considered. Our approach, based on t-statistics, is a powerful tool for visualizing system-level effects, but it serves as a proxy measure rather than a formal index of effect size. For clarity, in the primary (conservative) GEE specification clustered by Gene (N = 22), neither the Serotonin_System indicator nor the effect Magnitude reached statistical significance. Given the modest effect sizes and incomplete concordance between transcription and protein abundance, our conclusions are framed as suggestive and hypothesis-generating rather than definitive evidence of mechanism.

This work continues a line of systems and network studies in which integration of multi-level measures reveals coordinated molecular responses and their links to phenotype [35,57,58]. A population-averaged GEE analysis allows formal testing of these system-level patterns while accounting for clustering, thereby connecting the intuitive scatter plots of t-statistics with inferential statistics.

## 5. Conclusions

We integrated qPCR and Western blotting assays to show that chronic ethanol and 5-HT_7_ receptor overexpression operate through interacting, region-specific regulatory rules. When we mapped effects in the (t_1_, t_2_) space and applying population-averaged GEE, two patterns were observed: region-specific concordance, pronounced in the hippocampus and showing a trend in the hypothalamus; and level-specific divergence, where antagonistic transcriptional shifts are partially reconciled at the protein level, consistent with molecular buffering.

In the primary GEE model clustered by molecular marker (GENE = 22), neither the serotonergic system term nor overall effect magnitude reached significance. Using t-statistics as coordinates prioritizes effect direction and relative strength and should be interpreted alongside formal ANOVA/GEE inference.

These findings indicate that neuroadaptation to combined genetic and ethanol factors is governed by region- and level-specific rules rather than a single global program. As others have argued, data can become toxic in excess when structure is ignored. Recognizing this structure turns heterogeneous measurements into coherent evidence and clarifies where therapeutic modulation of 5-HT_7_/BDNF pathways is might be most effective.

## Figures and Tables

**Table 1:** The primer sequences, annealing temperatures, and amplicon lengths.

Gene	Protein Associated with the Gene	Nucleotide Sequence	Annealing Temperature	Product Length, bp
*Polr2a*	RPB1 subunit of DNA-directed RNA Polymerase II (Pol II)	F 5′-tgtgacaactccatacaatgc-3′ R 5′-ctctcttactgaatttgcgtact-3′	60	194
*Htr1a*	5-HT_1A_ receptor	F 5’-gactgccaccctctgccctatatc-3’ R 5’-tcagcaaggcaaacaattccag-3’	62	199
*Htr7*	5-HT_7_ receptor	F 5’-ggctacacgatctactccaccg-3’ R 5’-cgcacactcttccacctccttc-3’	65	198
*Htr2a*	5-HT_2A_ receptor	F 5′-agaagccaccttgtgtgtga-3′ R 5′-ttgctcattgctgatggact-3′	61	169
*Tph2*	TPH2	F 5′-cattcctcgcacaattccagtcg-3′ R 5′-cttgacatattcaactagacgctc-3′	61	236
*Slc6a4*	5-HTT	F 5′-cgctctactacctcatctcctcc-3′ R 5′-gtcctgggcgaagtagttgg-3′	63	101
*Bdnf*	BDNF	F 5′-tagcaaaaagagaattggctg-3′ R 5′-tttcaggtcatggatatgtcc-3′	59	255
*Ntrk2*	TrkB	F 5′-cattcactgtgagaggcaacc-3′ R 5′-atcagggtgtagtctccgttatt-3′	63	175
*Ngfr*	P75NTR	F 5′-acaacacccagcacccagga-3′ R 5′-cacaaccacagcagccaaga-3′	62	171
*Creb1*	CREB	F 5′-gctggctaacaatggtacggat-3′ R 5′-tggttgctgggcactagaat-3′	64	140

**Table 2:** List of antibodies used and immunodetection conditions.

Antibody Target	Catalogue Number	Dilution and Incubation Condition
5-HT_1A_ receptor	Abcam, Cambridge, UK, ab85615	1:1000, BSA 5% in TBST, 4 °C O/N
5-HT_7_ receptor	Abcam, Cambridge, UK, ab128892	1:1000, BSA 5% in TBST, 4 °C O/N
5-HT_2A_ receptor	Novus Biologicals, USA, Novus NBP1-49172	1:300, BSA 5% in TBST, 4 °C O/N
TPH2	Abcam, Cambridge, UK, ab184505	1:1000, BSA 5% in TBST, 4 °C O/N
5-HTT	USBiological Life Sciences, 303614	1:1000, BSA 5% in TBST, 4 °C O/N
TrkB receptor	Abcam, Cambridge, UK, ab18987	1:500, BSA 5% in TBST, 4 °C O/N
GAPDH	Abcam, Cambridge, UK, ab8245	1:10,000, BSA 5% in TBST, 4 °C O/N
BDNF	Abcam, Cambridge, UK, ab108319	1:1000, 5% BSA in TBST, 4 °C O/N
proBDNF	Santa Cruz, USA, sc-65514	1:250 with NaN_3_ addition in 5% BSA in TBST, 4 °C O/N
p75 receptor	Abcam, Cambridge, ab52987	1:2000, 5% goat milk in TBST, 4 °C O/N
CREB	Abcam, Cambridge, ab31387	1:1000, 5% BSA in TBST, 4 °C O/N
pCREB	Abcam, Cambridge, ab32096	1:1000, 5% BSA in TBST, 4 °C O/N
EGFP	LSBio (LifeSpan) Cat# LS-C50850-500,	1:1000, 5% milk in TBST, 4 °C O/N

**Table 3:** Full results of the primary GEE model and sensitivity analyses. This table presents the detailed outputs of the population-averaged models used to test the predictors of same-direction (synergistic) regulatory patterns. All models are based on the formula: EffectDirection ~ DataType + C(Region) + C(GeneSystem) + EffectMagnitude. Results are presented as both raw coefficients (log-odds) and Odds Ratios (OR) with 95% CIs. Primary Model: GEE with clustering by Gene (N = 22) and an Exchangeable working correlation. This conservative specification was used for the main inferences reported in the article. Reference levels: Data type = protein; Region = frontal cortex (FC); Gene system = 5-HT.

Predictor	β	SE	z	*p*-Value	95% CI for β
Intercept	−1.125	0.586	−1.919	0.055	−2.273 to 0.024
Data type: mRNA (vs. protein)	−1.068	0.484	−2.209	0.027	−2.016 to −0.121
Region: Hippocampus (vs. FC)	1.812	0.72	2.516	0.012	0.401 to 3.224
Region: Hypothalamus (vs. FC)	1.502	0.776	1.936	0.053	−0.018 to 3.022
Region: Midbrain (vs. FC)	1.232	0.849	1.451	0.147	−0.432 to 2.896
Gene system: 5-HT (vs. BDNF)	−0.192	0.466	−0.412	0.680	−1.105 to 0.721
EffectMagnitude	0.161	0.172	0.94	0.347	−0.175 to 0.498
Predictor	Odds ratio (OR)			*p*-value	95% CI (OR)
Intercept	0.325			0.055	0.103 to 1.025
Data type: mRNA (vs. protein)	0.344			0.027	0.133 to 0.886
Region: Hippocampus (vs. FC)	6.124			0.012	1.493 to 25.119
Region: Hypothalamus (vs. FC)	4.49			0.053	0.982 to 20.534
Region: Midbrain (vs. FC)	3.428			0.147	0.649 to18.108
Gene system: 5-HT (vs. BDNF)	0.825			0.68	0.331 to2.056
EffectMagnitude	1.175			0.347	0.839 to 1.645

## Data Availability

The datasets generated during the current study are available from the corresponding author on reasonable request.

## Supplementary Materials

The following supporting information can be downloaded at https://www.mdpi.com/article/10.3390/biom16010106/s1, Table S1. Summary of two-way ANOVA results for all individual molecular targets, Table S2. Effects of chronic alcoholization and 5-HT_7_ overexpression on behavioral outcomes, Supplementary Table S3. Per-target OLS model coefficients used for system-level visualization on mRNA data (from qPCR), Table S4. Per-target OLS model coefficients used for system-level visualization on protein data (from Western Blot), Supplementary Table S5. Full results of the primary GEE model and sensitivity analyses, Figure S1. Key predictors effects across different model specifications. Figure S2. Representative coronal midbrain section from the validation animal showing EGFP expression/spread (green) with DAPI counterstain (blue).

## Abbreviations

The following abbreviations are used in this manuscript:

BDNF	Brain-derived neurotrophic factor
5-HT	Serotonin
AUD	Alcohol use disorders
GEE	Generalized estimating equations
OLS	Ordinary Least Squares
VTA	Ventral tegmental area
