# Supplementary material for: Systemic Interplay of BDNF and Serotonin Pathways Defines Behavioral and Molecular Responses to Midbrain 5-HT7 Overexpression and Chronic Ethanol Consumption
Source: Biomolecules. 2026 Jan 8;16(1):106. doi: 10.3390/biom16010106 (PMC12838814; doi:10.3390/biom16010106)
Supplement: Supplementary file 1 [file biomolecules-16-00106-s001.zip › biomolecules-4029717-supplementary.pdf]

Supplementary Table S1. Summary of two-way ANOVA results for all individual molecular targets.

This table provides the detailed statistical output of the per-target two-way ANOVAs (factors: Ethanol, Overexpression, and their interaction). F-statistics and p-values are presented for each target across all four measured brain regions. These results form the basis for the subsequent system-level integrative analyses.

(a) qPCR data for mRNA abundance.

Gene	Factor	Midbrain	Fr.cortex	Hippocampus	Hypothalamus
Htr1a	Ethanol	F(1, 33) = 13.488<br>p<0.001	F(1, 33) = 49.647<br>p<0.001	F(1, 34) = 4.306<br>p=0.0456	F(1, 33) < 1
	Overexpression	F(1, 33) < 1	F(1, 33) < 1	F(1, 34) = 9.183<br>p=0.0046	F(1, 33) = 1.258<br>p=0.2701
	Ethanol*<br>Overexpression	F(1, 33) < 1	F(1, 33) < 1	F(1, 34) = 5.127<br>p=0.0300	F(1, 33) = 5.606<br>p=0.0239
Htr7	Ethanol	F(1, 33) = 5.786<br>p=0.0219	F(1, 33) < 1	F(1, 34) < 1	F(1, 32) = 3.240<br>p=0.0813
	Overexpression	F(1, 33) = 85.646<br>p<0.001	F(1, 33) < 1<br>F(1, 33) = 1.259	F(1, 34) < 1	F(1, 32) < 1
	Ethanol*<br>Overexpression	F(1, 33) = 5.915<br>p=0.0206	p=0.2699	F(1, 34) < 1	F(1, 32) < 1
Htr2a	Ethanol	F(1, 33) = 5.487<br>p=0.0253	F(1, 33) < 1	F(1, 31) < 1	F(1, 31) < 1
	Overexpression	F(1, 33) = 2.953<br>p=0.0951	F(1, 33) < 1	F(1, 31) = 1.592<br>p=0.2164	F(1, 31) = 4.311<br>p=0.0462
	Ethanol*<br>Overexpression	F(1, 33) = 2.609<br>p=0.1158	F(1, 33) = 1.525<br>p=0.2255	F(1, 31) = 1.019<br>p=0.3206	F(1, 31) = 3.408<br>p=0.0744
Bdnf	Ethanol	F(1, 33) = 1.869<br>p=0.1809	F(1, 32) = 21.245<br>p<0.001	F(1, 34) = 9.868<br>p=0.0035	F(1, 34) < 1
	Overexpression	F(1, 33) < 1	F(1, 32) < 1	F(1, 34) = 3.263<br>p=0.0797	F(1, 34) = 2.985<br>p=0.0931
	Ethanol*<br>Overexpression	F(1, 33) < 1	F(1, 32) = 1.902<br>p=0.1774	F(1, 34) < 1	F(1, 34) = 3.894<br>p=0.0566
Ntrk2	Ethanol	F(1, 33) = 9.730<br>p=0.0037	F(1, 33) < 1	F(1, 33) < 1	F(1, 31) = 43.710<br>p<0.001
	Overexpression	F(1, 33) < 1	F(1, 33) = 1.582<br>p=0.2174	F(1, 33) < 1	F(1, 31) = 7.981<br>p=0.0072
	Ethanol*<br>Overexpression	F(1, 33) < 1	F(1, 33) = 1.104<br>p=0.3010	F(1, 33) = 6.045<br>p=0.0194	F(1, 31) < 1
Ngfr	Ethanol	F(1, 33) = 2.001<br>p=0.1665	F(1, 28) = 12.105<br>p=0.0017	F(1, 32) < 1	F(1, 32) = 27.806<br>p<0.001
	Overexpression	F(1, 33) < 1	F(1, 28) = 3.553<br>p=0.0698	F(1, 32) = 2.484<br>p=0.1248	F(1, 32) < 1
	Ethanol*<br>Overexpression	F(1, 33) < 1	F(1, 28) = 2.268<br>p=0.1432	F(1, 32) < 1	F(1, 32) < 1
Creb1	Ethanol	F(1, 32) = 75.067<br>p<0.001	F(1, 33) = 4.830<br>p=0.0351	F(1, 34) = 2.467<br>p=0.1256	F(1, 33) = 6.063<br>p=0.0192
	Overexpression	F(1, 32) = 1.765<br>p=0.1934	F(1, 33) = 1.663<br>p=0.2061	F(1, 34) = 17.330<br>p<0.001	F(1, 33) = 2.040<br>p=0.1626
	Ethanol*<br>Overexpression	F(1, 32) < 1	F(1, 33) = 3.790<br>p=0.0601	F(1, 34) = 5.537<br>p=0.0245	F(1, 33) < 1
Tph2	Ethanol	F(1, 33) = 2.943<br>p=0.0956			
	Overexpression	F(1, 33) < 1			
	Ethanol*<br>Overexpression	F(1, 33) < 1			
Slc6a4	Ethanol	F(1, 33) = 1.433<br>p=0.2397			
	Overexpression	F(1, 33) < 1			
	Ethanol*<br>Overexpression	F(1, 33) < 1			

(b) Western Blot data for protein levels.

Gene	Factor	Midbrain	Fr.cortex	Hippocampus	Hypothalamus
5-HTR1A	Ethanol	$F(1, 21) < 1$	$F(1, 21) < 1$	$F(1, 20) = 3.781$<br>$p=0.0660$	$F(1, 21) = 4.798$<br>$p=0.0399$
	Overexpression	$F(1, 21) < 1$	$F(1, 21) < 1$	$F(1, 20) = 1.079$<br>$p=0.3113$	$F(1, 21) < 1$
	Ethanol* Overexpression	$F(1, 21) < 1$	$F(1, 21) < 1$	$F(1, 20) < 1$	$F(1, 21) = 1.045$<br>$p=0.3183$
5-HTR7	Ethanol	$F(1, 21) = 4.655$<br>$p=0.0427$	$F(1, 20) = 5.169$<br>$p=0.0342$	$F(1, 21) < 1$	$F(1, 20) < 1$
	Overexpression	$F(1, 21) < 1$	$F(1, 20) < 1$	$F(1, 21) < 1$	$F(1, 20) < 1$
	Ethanol* Overexpression	$F(1, 21) < 1$	$F(1, 20) < 1$	$F(1, 21) < 1$	$F(1, 20) < 1$
5-HTR2A	Ethanol	$F(1, 20) < 1$	$F(1, 21) < 1$	$F(1, 21) < 1$	$F(1, 21) = 2.25$<br>$p=0.1484$
	Overexpression	$F(1, 20) < 1$	$F(1, 21) = 2.881$<br>$p=0.1044$	$F(1, 21) = 1.615$<br>$p=0.2177$	$F(1, 21) < 1$
	Ethanol* Overexpression	$F(1, 20) = 1.11$<br>$p=0.3027$	$F(1, 21) = 2.376$<br>$p=0.1382$	$F(1, 21) = 1.836$<br>$p=0.1899$	$F(1, 21) < 1$
BDNF	Ethanol	$F(1, 20) = 1.174$<br>$p=0.2915$	$F(1, 18) < 1$	$F(1, 21) < 1$	$F(1, 20) = 1.458$<br>$p=0.2413$
	Overexpression	$F(1, 20) = 1.005$<br>$p=0.3281$	$F(1, 18) = 4.540$<br>$p=0.0471$	$F(1, 21) < 1$	$F(1, 20) < 1$<br>$F(1, 20) = 3.019$
	Ethanol* Overexpression	$F(1, 20) < 1$	$F(1, 18) = 1.598$<br>$p=0.2224$	$F(1, 21) < 1$	$p=0.0977$
proBDNF	Ethanol	$F(1, 21) < 1$	$F(1, 21) < 1$	$F(1, 20) < 1$	$F(1, 21) = 2.207$<br>$p=0.1523$
	Overexpression	$F(1, 21) = 5.959$<br>$p=0.0236$	$F(1, 21) = 2.938$<br>$p=0.1013$	$F(1, 20) = 2.108$<br>$p=0.1620$	$F(1, 21) = 1.908$<br>$p=0.1817$
	Ethanol* Overexpression	$F(1, 21) = 2.852$<br>$p=0.1061$	$F(1, 21) = 3.399$<br>$p=0.0794$	$F(1, 20) = 7.325$<br>$p=0.0136$	$F(1, 21) < 1$
proBDNF/BDNF	Ethanol	$F(1, 20) < 1$	$F(1, 20) < 1$	$F(1, 20) < 1$	$F(1, 20) < 1$
	Overexpression	$F(1, 20) < 1$	$F(1, 20) < 1$	$F(1, 20) = 1.562$<br>$p=0.2258$	$F(1, 20) < 1$
	Ethanol* Overexpression	$F(1, 20) < 1$	$F(1, 20) < 1$	$F(1, 20) = 3.178$<br>$p=0.0898$	$F(1, 20) = 5.466$<br>$p=0.0299$
P75NTR	Ethanol	$F(1, 18) = 2.322$<br>$p=0.1450$	$F(1, 21) < 1$	$F(1, 20) = 1.002$<br>$p=0.3288$	$F(1, 20) = 6.518$<br>$p=0.0190$
	Overexpression	$F(1, 18) = 6.545$<br>$p=0.0198$	$F(1, 21) < 1$	$F(1, 20) < 1$	$F(1, 20) < 1$
	Ethanol* Overexpression	$F(1, 18) < 1$	$F(1, 21) < 1$	$F(1, 20) < 1$	$F(1, 20) < 1$
TrkB	Ethanol	$F(1, 21) = 1.581$<br>$p=0.2224$	$F(1, 19) < 1$	$F(1, 21) < 1$	$F(1, 21) < 1$
	Overexpression	$F(1, 21) < 1$	$F(1, 19) < 1$<br>$F(1, 19) = 6.030$	$F(1, 21) < 1$<br>$F(1, 21) = 1.110$	$F(1, 21) < 1$
	Ethanol* Overexpression	$F(1, 21) = 1.885$<br>$p=0.1843$	$p=0.0239$	$p=0.3041$	$F(1, 21) < 1$
CREB	Ethanol	$F(1, 21) = 3.947$<br>$p=0.0602$	$F(1, 21) < 1$	$F(1, 20) < 1$	$F(1, 19) < 1$
	Overexpression	$F(1, 21) = 2.506$<br>$p=0.1284$	$F(1, 21) < 1$	$F(1, 20) < 1$	$F(1, 19) < 1$
	Ethanol* Overexpression	$F(1, 21) = 1.862$<br>$p=0.1869$	$F(1, 21) < 1$	$F(1, 20) < 1$	$F(1, 19) < 1$
p-CREB	Ethanol	$F(1, 19) < 1$	$F(1, 21) < 1$	$F(1, 20) = 1.514$<br>$p=0.2328$	$F(1, 20) < 1$
	Overexpression	$F(1, 19) = 4.981$<br>$p=0.0379$	$F(1, 21) = 1.201$<br>$p=0.2856$	$F(1, 20) < 1$	$F(1, 20) < 1$

	Ethanol* Overexpression	F(1, 19) = 4.797 p=0.0412	F(1, 21) = 1.070 p=0.3127	F(1, 20) < 1	F(1, 20) < 1
pCREB/CREB	Ethanol	F(1, 20) = 1.204 p=0.2856	F(1, 20) < 1	F(1, 19) < 1	F(1, 21) = 2.259 p=0.1477
	Overexpression	F(1, 20) = 2.340 p=0.1417	F(1, 20) < 1	F(1, 19) = 1.058 p=0.3166	F(1, 21) = 2.100 p=0.1621
	Ethanol* Overexpression	F(1, 20) = 1.589 p=0.2220	F(1, 20) < 1	F(1, 19) = 1.682 p=0.2101	F(1, 21) = 1.354 p=0.2577
TPH2	Ethanol	F(1, 19) < 1			
	Overexpression	F(1, 19) = 1.021 p=0.3249			
	Ethanol* Overexpression	F(1, 19) < 1			
5-HTT	Ethanol	F(1, 20) = 5.464 p=0.0299			
	Overexpression	F(1, 20) < 1			
	Ethanol* Overexpression	F(1, 20) < 1			

Supplementary Table S2. Effects of chronic alcoholization and 5-HT7 overexpression on behavioral outcomes.

This table summarizes the results of the two-way ANOVAs for the behavioral tests conducted. F-statistics and p-values are presented for the main effects of Alcohol, Overexpression, and their interaction on outcomes from the Open Field Test, Forced Swim Test, Novel Object Recognition task, and Dark-Light Box Test.

	**Alcohol**	**Overexpression**	**Alcohol* Overexpression**
Open Field Test (distance traveled, m)	F (1, 33) = 0,2491 P=0,6210	F (1, 33) = 0,6599 P=0,4224	F (1, 33) = 0,6713 P=0,4185
Forced Swim Test (mobility, %)	F (1, 27) = 17,37 P=0,0003	F (1, 27) = 1,968 P=0,1721	F (1, 27) = 1,466 P=0,2365
Novel Object Recognition (novel object preference, %)	F (1, 33) = 0,7655 P=0,3879	F (1, 33) = 1,730 P=0,1975	F (1, 33) = 0,2169 P=0,6445
Dark-Light Box Test (time spent in enlighted compartment, s)	F (1, 33) = 20,11 P<0,0001	F (1, 33) = 14,16 P=0,0007	F (1, 33) = 0,3280 P=0,5708
Dark-Light Box Test (number of head peaks toward enlighted compartment)	F (1, 32) = 24,10 P<0,0001	F (1, 32) = 0,007769 P=0,9303	F (1, 32) = 1,513 P=0,2277
Dark-Light Box Test (explored enlighted compartment, %)	F (1, 34) = 5,098 P=0,0305	F (1, 34) = 6,017 P=0,0195	F (1, 34) = 0,5443 P=0,4657

Supplementary Table S3. Per-target OLS model coefficients used for system-level visualization on mRNA data (from qPCR). This table provides the detailed output of the ordinary least squares (OLS) models fitted for each individual target–region pair. The model estimated the main effects of Chronic Alcoholization and 5-HT7 Overexpression. The resulting coefficients (β), their standard errors (SE), and the corresponding t-statistics (t = β/SE) are presented. These t-statistics were used as coordinates for the exploratory scatter plots shown in Figure 7.

		**Chronic alcoholization**			**5-HT7 overexpression**		
**Gene**	**Region**	**β**	**SE**	**t**	**β**	**SE**	**t**
Creb1	Frontal cortex	7,7768	3,6809	2,1128	-4,5502	3,6701	-1,2398
Bdnf		9,1845	2,0197	4,5475	-0,8721	2,0197	-0,4318
Ngfr		0,4272	0,1255	3,4055	0,2274	0,1232	1,8451
Ntrk2		3,1421	30,3248	0,1036	-37,9669	30,236	-1,2557
Htr1a		-18,599	2,6191	-7,1013	-1,5696	2,6114	-0,601
Htr2a		1,4241	5,9786	0,2382	-2,9058	5,9611	-0,4875
Htr7		0,2606	0,5414	0,4814	-0,1826	0,5399	-0,3382
Creb1	Hippocampus	3,626	2,4538	1,4777	-9,6114	2,4538	-3,9169
Bdnf		-10,0278	3,1687	-3,1646	-5,7667	3,1687	-1,8199
Ngfr		-0,6636	1,0988	-0,604	1,7452	1,0971	1,5907
Ntrk2		11,9927	16,6015	0,7224	8,3266	16,5529	0,503
Htr1a		-6,3838	3,2527	-1,9626	-9,3224	3,2527	-2,866
Htr2a		-0,1121	1,4428	-0,0777	-1,8202	1,4428	-1,2615
Htr7		2,3434	2,8447	0,8238	-1,5894	2,8447	-0,5587
Creb1	Hypothalamus	12,1695	4,9222	2,4724	7,0375	4,9078	1,4339
Bdnf		-0,1313	1,9221	-0,0683	3,1915	1,9221	1,6605
Ngfr		-3,8507	0,7299	-5,2755	0,5136	0,7345	0,6993
Ntrka2		-338,9644	54,5959	-6,2086	104,4114	54,9361	1,9006
Htr1a		-1,0395	2,2854	-0,4549	2,3986	2,2787	1,0526
Htr2a		0,5812	2,0247	0,2871	4,0673	2,0313	2,0023
Htr7		-2,6134	3,9776	-0,657	5,914	3,9776	1,4868
Creb1	Midbrain	21,8578	2,489	8,7817	3,3463	2,4852	1,3465
Bdnf		1,4419	1,0407	1,3855	-0,7664	1,0407	-0,7365
Ngfr		-1,5008	1,0565	-1,4205	-0,0456	1,0565	-0,0431
Ntrk2		-100,4178	31,7659	-3,1612	31,7951	31,7659	1,0009
Htr1a		11,7191	3,175	3,691	-0,087	3,175	-0,0274
Htr2a		1,7662	0,7717	2,2888	-1,2957	0,7717	-1,6791
Htr7		-295,5687	131,4581	-2,2484	1137,1526	131,4581	8,6503
Tph2		22,5715	12,9721	1,74	5,6199	12,9721	0,4332
Slc6a4		2,9965	2,467	1,2146	-2,0422	2,467	-0,8278

Supplementary Table S4. Per-target OLS model coefficients used for system-level visualization on protein data (from Western Blot). This table provides the detailed output of the ordinary least squares (OLS) models fitted for each individual target–region pair. The model estimated the main effects of Chronic Alcoholization and 5-HT7 Overexpression. The resulting coefficients (β), their standard errors (SE), and the corresponding t-statistics (t = β/SE) are presented. These t-statistics were used as coordinates for the exploratory scatter plots shown in Figure 8.

		**Chronic alcoholization**			**5-HT7 overexpression**		
**Protein**	**Region**	**β**	**SE**	**t**	**β**	**SE**	**t**
5-HT1A	Frontal cortex	16,7757	19,1492	0,8761	-0,2463	19,2731	-0,0128
5-HT2A		21,3292	22,4497	0,9501	-35,5268	22,595	-1,5723
5-HT7		30,7468	13,2963	2,3124	5,3476	13,3427	0,4008
BDNF		-10,8896	21,2129	-0,5133	24,3781	21,3502	1,1418
proBDNF		-12,3509	24,4091	-0,506	39,9833	24,5671	1,6275
P75		-14,8181	18,76	-0,7899	-1,7911	18,8814	-0,0949
TrkB		-1,2053	23,5449	-0,0512	13,4129	23,5449	0,5697
CREB		3,9292	10,4748	0,3751	-7,8014	10,5426	-0,74
pCREB		-14,6432	24,2064	-0,6049	26,6534	24,3631	1,094
pCREB/CREB		-0,0137	0,1509	-0,0906	0,1379	0,1531	0,9007
proBDNF/BDNF		-0,0076	0,209	-0,0362	0,0645	0,2098	0,3075
5-HT1A	Hippocampus	17,3366	12,4476	1,3928	-17,3801	12,5282	-1,3873
5-HT2A		4,8513	7,7708	0,6243	10,3009	7,8211	1,3171
5-HT7		4,0516	24,6624	0,1643	5,886	24,8221	0,2371
BDNF		14,5621	15,2888	0,9525	-1,7021	15,3877	-0,1106
proBDNF		7,6889	23,7616	0,3236	17,6986	23,9154	0,74
P75		29,6694	28,9717	1,0241	24,1498	29,0728	0,8307
TrkB		1,3799	16,7388	0,0824	-12,1197	16,8471	-0,7194
CREB		-10,773	16,4829	-0,6536	-13,1604	16,5404	-0,7956
pCREB		-25,2105	19,9963	-1,2608	-0,3266	19,9963	-0,0163
pCREB/CREB		-0,306	0,6648	-0,4603	0,6724	0,6648	1,0114
proBDNF/BDNF		0,0213	0,0735	0,2895	0,0878	0,0738	1,1897
5-HT1A	Hypothalamus	134,9635	61,6791	2,1882	23,6913	62,0783	0,3816
5-HT2A		-21,3248	13,5609	-1,5725	9,1347	13,6486	0,6693
5-HT7		0,0668	0,0947	0,7055	0,0713	0,0951	0,7495
BDNF		-56,797	71,4925	-0,7944	-12,8564	71,9552	-0,1787
proBDNF		3,6453	2,4002	1,5187	3,4117	2,4158	1,4123

P75		8,672	3,3205	2,6117	1,6225	3,3205	0,4886
TrkB		0,0064	0,1203	0,0534	0,0045	0,1211	0,0373
CREB		-0,0027	0,0795	-0,0337	0,0365	0,0795	0,4596
pCREB		0,0061	0,0094	0,6424	-0,0019	0,0095	-0,2026
pCREB/CREB		0,2285	0,1532	1,4912	0,2217	0,1542	1,4375
proBDNF/BDNF		0,0085	0,0119	0,7137	0,0022	0,0119	0,1868
5-HT1A	Midbrain	-13,998	26,1449	-0,5354	13,7267	26,3141	0,5216
5-HT2A		-2,4864	6,3245	-0,3931	-0,1838	6,3466	-0,029
5-HT7		41,7036	19,2741	2,1637	-14,2721	19,3989	-0,7357
5-HTT		-47,679	20,0343	-2,3799	-8,9893	20,2478	-0,444
TPH2		2,1055	14,2718	0,1475	-15,1202	14,6076	-1,0351
BDNF		12,0331	35,589	0,3381	54,5207	35,8193	1,5221
proBDNF		4,8168	52,0445	0,0926	122,8057	52,3814	2,3445
P75		-37,7131	19,0015	-1,9847	10,9983	19,0015	0,5788
TrkB		-10,3234	8,3739	-1,2328	-3,2534	8,4281	-0,386
CREB		-39,0572	20,0416	-1,9488	-31,3217	20,1713	-1,5528
pCREB		0,0056	0,0081	0,6839	0,0166	0,0081	2,0459
pCREB/CREB		-0,1283	0,1185	-1,0822	0,1795	0,119	1,5088
proBDNF/BDNF		-0,2468	0,7744	-0,3187	-0,467	0,7826	-0,5967

Supplementary Table S5. Full results of the primary GEE model and sensitivity analyses.

This table presents the detailed outputs of the population-averaged models used to test the predictors of same-direction (synergistic) regulatory patterns. All models are based on the formula: EffectDirection ~ DataType + C(Region) + C(GeneSystem) + EffectMagnitude. Results are presented as both raw coefficients (log-odds) and Odds Ratios (OR) with 95% CIs.

(a) Primary Model: GEE with clustering by Gene (N=22) and an Exchangeable working correlation. This conservative specification was used for the main inferences reported in the article.

	**coef**	**std err**	**z**	**P>|z|**	**[0.025**	**0.975]**
Intercept	-1.1246	0.586	-1.919	0.055	-2.273	0.024
DataType[T.mRNA]	-1.0683	0.484	-2.209	0.027	-2.016	-0.121
C(Region)[T.Hippocampus]	1.8122	0.72	2.516	0.012	0.401	3.224
C(Region)[T.Hypothalamus]	1.5019	0.776	1.936	0.053	-0.018	3.022
C(Region)[T.Midbrain]	1.232	0.849	1.451	0.147	-0.432	2.896
C(GeneSystem)[T.Serotonin_System]	-0.1918	0.466	-0.412	0.68	-1.105	0.721
EffectMagnitude	0.1613	0.172	0.94	0.347	-0.175	0.498

	**OR**	**CI_low**	**CI_up**	**p**
Intercept	0.325	0.103	1.025	0.055
DataType[T.mRNA]	0.344	0.133	0.886	0.027
C(Region)[T.Hippocampus]	6.124	1.493	25.119	0.012
C(Region)[T.Hypothalamus]	4.49	0.982	20.534	0.053
C(Region)[T.Midbrain]	3.428	0.649	18.108	0.147
C(GeneSystem)[T.Serotonin_System]	0.825	0.331	2.056	0.68
EffectMagnitude	1.175	0.839	1.645	0.347

(b) Sensitivity Analysis 1 (Correlation Structure): GEE with clustering by Gene (N=22) and an Independence working correlation. Results confirm the robustness of the primary model to the choice of correlation structure.

	**coef**	**std err**	**z**	**P>|z|**	**[0.025**	**0.975]**
Intercept	-1.1447	0.593	-1.93	0.054	-2.307	0.018
DataType[T.mRNA]	-1.0682	0.477	-2.241	0.025	-2.003	-0.134
C(Region)[T.Hippocampus]	1.8271	0.719	2.541	0.011	0.418	3.236
C(Region)[T.Hypothalamus]	1.5164	0.77	1.97	0.049	0.008	3.025
C(Region)[T.Midbrain]	1.2352	0.845	1.463	0.144	-0.42	2.89
C(GeneSystem)[T.Serotonin_System]	-0.1747	0.47	-0.372	0.71	-1.095	0.746
EffectMagnitude	0.1669	0.176	0.95	0.342	-0.177	0.511

	**OR**	**CI_low**	**CI_up**	**p**
Intercept	0.318	0.1	1.018	0.054
DataType[T.mRNA]	0.344	0.135	0.875	0.025
C(Region)[T.Hippocampus]	6.216	1.519	25.44	0.011
C(Region)[T.Hypothalamus]	4.556	1.008	20.599	0.049
C(Region)[T.Midbrain]	3.439	0.657	18.001	0.144
C(GeneSystem)[T.Serotonin_System]	0.84	0.335	2.108	0.71

EffectMagnitude	1.182	0.837	1.667	0.342

(c) Sensitivity Analysis 2 (Clustering Unit): GLM with cluster-robust standard errors grouped by GeneEntity (N=10). This specification confirms the stability of key findings when using a biologically unified clustering approach.

	**coef**	**std err**	**z**	**P>|z|**	**[0.025**	**0.975]**
Intercept	-1.1447	0.976	-1.172	0.241	-3.058	0.769
DataType[T.mRNA]	-1.0682	0.53	-2.015	0.044	-2.107	-0.029
C(Region)[T.Hippocampus]	1.8271	0.892	2.049	0.04	0.079	3.575
C(Region)[T.Hypothalamus]	1.5164	0.861	1.761	0.078	-0.172	3.204
C(Region)[T.Midbrain]	1.2352	1.023	1.207	0.227	-0.771	3.241
C(GeneSystem)[T.Serotonin_System]	-0.1747	0.233	-0.749	0.454	-0.632	0.283
EffectMagnitude	0.1669	0.208	0.803	0.422	-0.241	0.575

	**OR**	**CI_low**	**CI_up**	**p**
Intercept	0.318	0.047	2.157	0.241
DataType[T.mRNA]	0.344	0.122	0.971	0.044
C(Region)[T.Hippocampus]	6.216	1.083	35.685	0.04
C(Region)[T.Hypothalamus]	4.556	0.842	24.641	0.078
C(Region)[T.Midbrain]	3.439	0.463	25.56	0.227
C(GeneSystem)[T.Serotonin_System]	0.84	0.531	1.327	0.454
EffectMagnitude	1.182	0.786	1.776	0.422

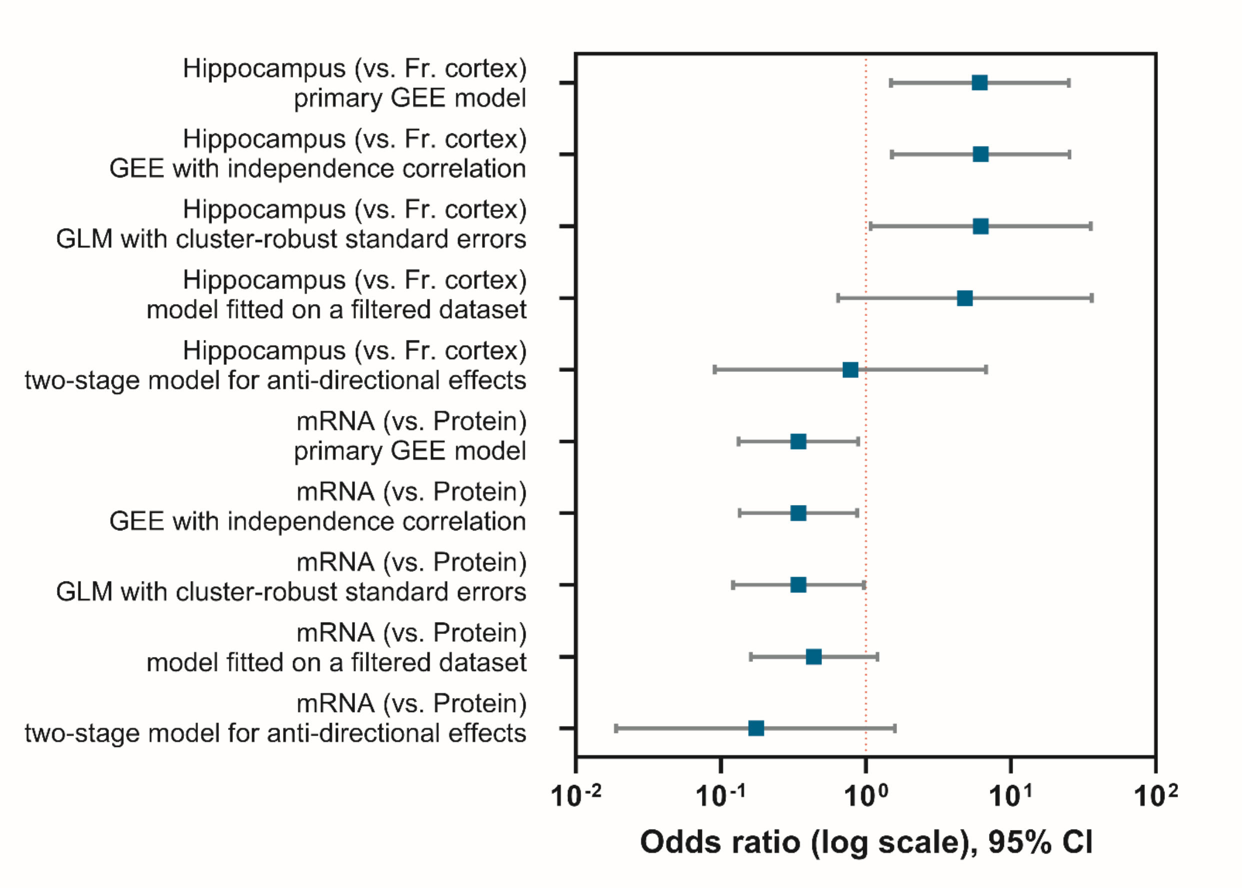

Supplementary Figure S1 Key predictors effects across different model specifications.

This forest plot illustrates the stability of the main effects for DataType (mRNA vs. Protein) and Region (Hippocampus vs. Frontal Cortex) across five different model specifications. Specifications include the primary GEE model (GEE_Exch), a GEE with independence correlation (GEE_Indep), a GLM with cluster-robust standard errors (GLM_cluster), a model fitted on a filtered dataset (Sens_A), and the two-stage model for anti-directional effects (Quadrant_anti). Despite variations in point estimates and confidence intervals, the direction of the effects remains consistent: the Odds Ratio for mRNA is consistently below 1.0, while the Odds Ratio for Hippocampus is consistently above 1.0, confirming the robustness of these findings.

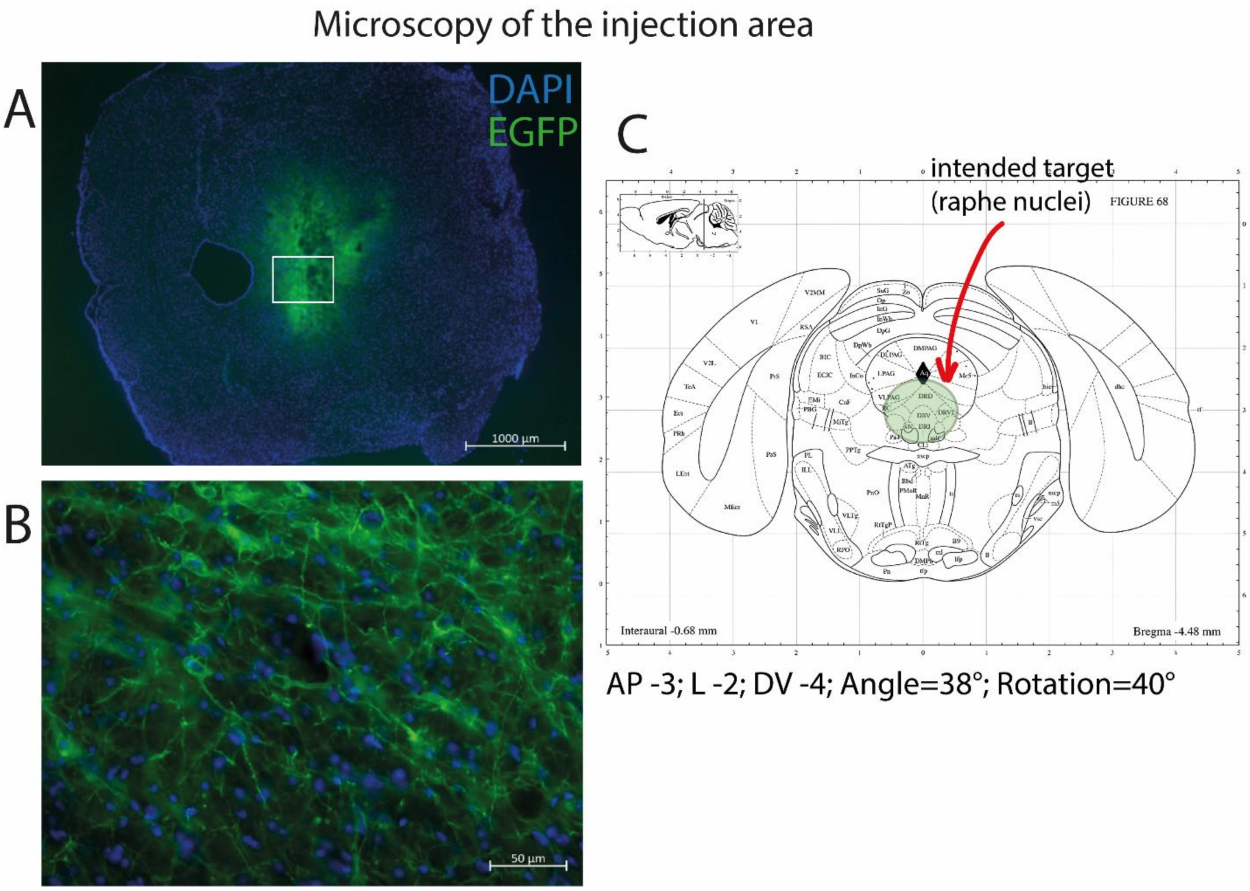

Supplementary Figure S2. Representative coronal midbrain section from the validation animal showing EGFP expression/spread (green) with DAPI counterstain (blue). Scale bar, (A) 1000 µm, (B); 50 µm (C) Targeted injection coordinates and intended raphe target shown on atlas section. Approach angle and rotation are indicated; Atlas template adapted from Paxinos and Franklin.
