# Supplementary material for: Systemic Interplay of BDNF and Serotonin Pathways Defines Behavioral and Molecular Responses to Midbrain 5-HT7 Overexpression and Chronic Ethanol Consumption
Source: Biomolecules. 2026 Jan 8;16(1):106. doi: 10.3390/biom16010106 (PMC12838814; doi:10.3390/biom16010106)
Supplement: Supplementary file 1 [file biomolecules-16-00106-s001.zip › biomolecules-4029717-original-images.zip!/raw_annotated_WB/WB_Membranes_Source_Data_Description.docx]

**The archive contains the following items:**

1. Raw Western blot images (per membrane)
   - All original scans for each membrane used in the study, with no rearrangement of lanes.
   - Only minimal processing has been applied where necessary (uniform adjustment of brightness/contrast applied to the entire image, and cropping to remove empty areas outside the gel/blot).
2. Region-specific annotated reference blots
   - For each brain region, one representative blot is provided with explicit lane annotations indicating the order of experimental groups and controls.
   - These reference blots are intended to illustrate the loading scheme and group order for that region.
3. Lane mapping table (Excel file)
   - A spreadsheet listing, for every membrane, the exact lane order and sample identity (brain region, group/treatment, animal or sample ID, and any excluded lanes).
   - Each row links a given lane to the corresponding raw image file name and to the figure/panel in the main manuscript.

**For each brain region, all membranes were run and loaded using an identical and fixed lane order (same sequence of experimental groups/controls). Therefore:**

- One fully annotated reference blot per region is sufficient to document the loading scheme and group order for that region.
- All remaining membranes for that region follow the same lane order, which is explicitly described in the Excel mapping table.

We deliberately chose not to overlay full textual labels on every raw blot in order to:

- avoid repetitive manual editing of 60+ files, which increases the risk of human error and inconsistent labelling;
- prevent excessive visual clutter and overlap of text with bands on high-density blots;
- keep the raw images as close as possible to the original data generated by the imaging system.

Despite using only one annotated example per region, each lane in every blot can be unambiguously identified:

- The raw image file name and lane index (counting from left to right) uniquely identify a band on a given membrane.
- The Excel lane mapping table gives, for each membrane and lane:
  - region,
  - group/treatment,
  - sample/animal ID,
  - and correspondence to the figure/panel in the manuscript.

This guarantees that reviewers and readers can:

- reproduce the exact mapping from any band in a figure back to the original raw image, and
- verify the grouping and assignment of each lane without ambiguity.

**We confirm that:**

- No bands were duplicated, moved, or selectively removed.
- Only uniform, global adjustments (brightness/contrast) were applied, and no nonlinear or local image manipulations were used.

If required, we are prepared to provide additional per-membrane overlays or alternative annotated versions of specific blots upon request, using the same lane mapping scheme described above.
